# Digital Health Interventions in Emergency Obstetric and Newborn Care Services in Low- and Middle-Income Countries: Scoping Review

**DOI:** 10.2196/75738

**Published:** 2025-10-28

**Authors:** Ni Putu Shartyanie, Intan Noor Hanifa, Nushrat Khan

**Affiliations:** 1 Population, Policy and Practice, Great Ormond Street Institute of Child Health, University College London, London, United Kingdom; 2 Universitas Islam Indonesia, Yogyakarta, Indonesia; 3 Department of Primary Care and Public Health, Imperial College London, 90 Wood Lane, London, W12 0BZ, United Kingdom, 44 (0) 207 5943 368

**Keywords:** digital health, digital health interventions, emergency obstetric and newborn care, maternal emergency, neonatal emergency, low-resource settings, low- and middle-income countries

## Abstract

**Background:**

The majority of global maternal and newborn deaths occur in low- and middle-income countries (LMICs), often due to a lack of resources, inadequate training of health care providers, and delayed or untimely care. Low-cost digital health interventions (DHIs) may help improve emergency obstetric and newborn care (EmONC) services in resource-limited settings by incorporating innovative approaches to enhance traditional models of care.

**Objective:**

This study aimed to systematically explore the key characteristics and usefulness of DHIs implemented for improving EmONC services in low-resource settings, as well as to identify barriers to implementation, given the importance of developing, implementing, and evaluating context-specific digital interventions for such settings.

**Methods:**

We followed the existing guidelines for conducting this scoping review, including the methodological framework for scoping studies, the updated Joanna Briggs Institute Methodology for Scoping Review, and the PRISMA-ScR (Preferred Reporting Items for Systematic Reviews and Meta-Analyses Extension for Scoping Reviews) guidelines. We searched 3 databases—PubMed, Web of Science, and the Cochrane Library—and identified studies published before November 2024 that described digital interventions aimed at enhancing EmONC in LMICs. Extracted data included the following: purposes, features, and functionalities of DHIs, mode of delivery, outcomes, and barriers to implementation. We used the Mixed Methods Appraisal Tool for assessing study quality.

**Results:**

A total of 33 eligible studies from 18 countries were included in the review that described 21 distinct DHIs. Most qualitative (7/8) and mixed methods studies (4/5) were of high quality. However, most quantitative descriptive studies (15/20) had some form of sampling issues. The digital interventions were reported either as standalone interventions (n=19) or combined with other nondigital approaches (n=13). Most studies used mobile health–based interventions, primarily targeting health care providers (n=28) through mobile apps and text-based messaging, with a focus on EmONC education and training (n=19). The review’s findings suggest generally positive impacts of DHIs on health care providers’ clinical practices, although maternal and perinatal health outcomes varied depending on the type of intervention. Although DHIs have the potential to improve services and access to EmONC in various health care settings, the advancement and implementation of these technologies in LMICs have progressed at a slow pace. The most common barrier identified was the lack of EmONC resources such as medication, skilled workforce, and ambulances, which challenged the implementation of these interventions.

**Conclusions:**

Our findings highlight the potential of DHIs to improve EmONC services in resource-scarce settings. Future research is needed in this area, which should prioritize the rigorous evaluation of DHIs, focusing on maternal and perinatal health outcomes, addressing context-specific challenges in health infrastructure, and evaluating the cost-effectiveness to support the development, effective use, and regulation of DHIs in LMICs. The proposed framework, based on our findings, can be used as a guide to develop and implement DHIs for EmONC support in low-resource settings.

## Introduction

Globally, about 280,000 women lost their lives due to pregnancy-related complications in 2020 [1]. In addition, the latest report indicates that the global newborn mortality rate remains high, with 2.3 million newborns dying every year within the first 28 days of life [2]. A report by the United Nations Inter-Agency Group for Child Mortality Estimation (UN IGME) highlighted that the 10 countries with the highest rates of global maternal deaths, stillbirths, and newborn deaths are primarily located in low- and middle-income countries (LMICs) in Central-Southern Asia and Sub-Saharan Africa [2,3].

To improve and standardize medical care during pregnancy and childbirth, the essential obstetric care (EOC) framework was introduced in 1997. This was updated in 2009 to include basic neonatal resuscitation [4-6] and has since evolved into the emergency obstetric and newborn care (EmONC) framework, which targets major direct obstetric complications and neonatal asphyxia as the primary newborn complication [5]. Although technical guidelines for adopting EmONC exist [5,7], LMICs continue to face challenges in its implementation, usage, and coverage, resulting in stagnated progress in global maternal and neonatal mortality reduction since 2010 [1,3,8,9]. The data further emphasize the urgent need for innovative strategies to prevent maternal and neonatal deaths in these regions.

Low-cost and context-specific digital health interventions (DHIs) could offer potential solutions to improve EmONC and address preventable causes of maternal and neonatal deaths in such settings [10,11]. Classifying DHIs by their intended purpose helps researchers and stakeholders better prioritize and use these technologies in this field. Using the World Health Organization (WHO) Classification of Digital Interventions, Services, and Applications in Health (CDISAH), DHIs can be classified into four groups according to their targeted primary users: (1) DHIs for persons, (2) DHIs for health care providers, (3) DHIs for health management and support personnel, and (4) DHIs for data services [12]. The classification can facilitate more effective integration of DHIs into health care systems by clarifying which specific functions within EmONC service delivery are intended to be addressed. For example, technologies such as wearable sensors and devices to track the health of mothers, fetuses, and newborns [13,14] can be categorized as person-focused DHIs, specifically serving the purpose of personal health tracking.

Furthermore, recent reviews of DHIs in maternal and child health suggest a growing use of digital technologies to support care for mothers and children in LMICs [10,11,15-17]. The majority of these interventions have focused on improving antenatal care access and participation, as well as promoting newborn immunization and breastfeeding as preventative measures [11]. Mobile health (mHealth) approaches [18], such as text messaging and smartphone apps in particular, have been shown to effectively boost antenatal care attendance and improve the timeliness of childhood immunizations in LMICs [10,15]. This indicates positive progress in adapting technologies in this field, although the number of studies on DHIs in antenatal care conducted in LMICs still lags behind those in high-income countries [19]. In addition, while these studies predominantly focus on antenatal care in preventing maternal and neonatal complications, they offer limited insight into how DHIs can support the full spectrum of EmONC.

In general emergency contexts, DHIs can improve care delivery and emergency response management in remote regions, such as through telemedicine to receive specialist consultations [20] or e-learning platforms and virtual simulations to improve health care providers’ skills without formal training [21]. Several scoping reviews have explored the use of DHIs in relation to specific emergency obstetric and neonatal contexts in LMICs, such as investigating the role of DHIs in triaging obstetric emergencies in maternity units [22], supporting pregnant women at high risk of pre-eclampsia and eclampsia [23], and managing neonatal emergency care [24]. Horiuchi et al [25] specifically reviewed 35 studies and found that e-learning platforms and mobile apps were commonly used tools for neonatal resuscitation training in LMICs. However, these reviews focused on improving maternal and neonatal emergency care services independently, treating them as different areas of focus. It is important to recognize that maternal emergencies, such as hemorrhage or eclampsia, can have direct consequences for the newborn, and vice versa. Therefore, adopting a comprehensive approach during labor, delivery, and the immediate postpartum period is more effective and efficient [5].

To our knowledge, no literature review has comprehensively explored the use of digital technologies for improving EmONC services, specifically in low-resource settings. The EmONC functions shall be understood as a continuum of care that connects maternal and newborn health, along with routine care and management of complications [6], highlighting the need for these components to be addressed in an integrated manner. Therefore, this scoping review aims to (1) systematically explore existing literature to identify the key features and functionalities of DHIs for improving access to and quality of EmONC services implemented in resource-poor settings and (2) analyze barriers to and advantages of implementing such interventions in similar settings.

## Methods

### Study Design

A scoping review approach was used due to the heterogeneous and emerging nature of research on DHIs aimed at enhancing the quality of and access to EmONC in LMICs [26]. This review was conducted following the methodological framework for scoping studies by Arksey and O’Malley [27], the updated Joanna Briggs Institute Methodology for Scoping Reviews [28,29], and the PRISMA-ScR (Preferred Reporting Items for Systematic Reviews and Meta-Analyses Extension for Scoping Reviews) standards [30]. The protocol for this scoping review was registered on the Open Science Framework [31].

### Eligibility Criteria

We included studies that described the application of digital technologies to enhance access to and quality of EmONC, published up to November 2024. All types of published primary study designs (randomized controlled trial [RCT], non-RCT, quasi-RCT, cohort, case-control, and qualitative studies). Project protocols, conference abstracts, and studies without full-text availability were excluded. There were no language restrictions as long as an English translation could be obtained. According to the Joanna Briggs Institute guidelines for scoping reviews, the Population-Concept-Context framework was used to formulate the research question and define the eligibility criteria:

1. Population: pregnant and postpartum women, newborns, or health care providers. Health care providers included community health workers (CHWs), as well as doctors, nurses, and midwives, due to their essential role in primary health care delivery in LMICs.
2. Concept: DHIs focused on improving access to or quality of emergency care for mothers and newborns during pregnancy or after labor and delivery. The interventions should aim to enhance the basic or comprehensive EmONC signal functions outlined by WHO [5]. Studies included could use digital technologies alone or in combination with other types of interventions.
3. Context: studies conducted in LMICs, as defined by the World Bank’s 2024 fiscal year income classification [32]. DHIs could be implemented in various health care settings.

### Data Sources, Search Strategy, and Study Selection

We searched the following 3 databases: PubMed, Web of Science, and the Cochrane Library. Search strategies using keywords and Medical Subject Headings terms were developed in consultation with a librarian to meet the specified criteria. The primary keywords used were “Digital health interventions” combined with “Emergency obstetric and newborn care” and “Low- and middle-income countries” (refer to Multimedia Appendix 1 for detailed search strategies for each database). We used the Rayyan (Robert Ayan of Rayyan Systems) software [33] for duplicate removal, as well as title and abstract screening. Two reviewers (NPS and INH) independently assessed the studies against their eligibility criteria—initially by titles and abstracts, followed by full-text screening of those that met the criteria. Before formal screening, the eligibility criteria were piloted on a sample of 10 titles and abstracts to ensure agreement. Any disagreements were resolved through discussion between the first author (NPS) and the senior team member (NK). In addition, we searched the reference lists of selected studies and gray literature using Google advanced search and focused queries on company websites and professional organizations to locate relevant reports, guidelines, and non–peer-reviewed materials. The supplementary searches applied the same search query to identify additional documents that fulfilled all inclusion criteria.

### Data Charting and Reporting the Results

Data extraction was conducted using Microsoft Excel following a predetermined data extraction form. The 2 reviewers (NPS and INH) independently extracted the following data: first author name, publication year, study design, country, setting, study aims, name and type of the DHIs implemented, duration of implementation, key findings regarding the usefulness, and barriers and advantages associated with the intervention. The extracted data were summarized descriptively and categorized based on the types of DHIs according to the WHO CDISAH classification and their intended user groups. Studies were classified as “exclusive” if they solely delivered digital interventions and “blended” if digital technology was used alongside other methods. Thematic analysis was applied to categorize the limitations of implementing DHIs in this field. Attempts were made to obtain unclear or incomplete data by emailing the investigators. Finally, all extracted data items were analyzed and categorized by the first author, and the results were verified by the last author.

The study quality assessment was conducted by NPS and INH using the 2018 version of the Mixed Methods Appraisal Tool—a tool designed to evaluate the quality of qualitative, quantitative, and mixed methods studies [34]. Disagreements were resolved between the 2 reviewers. In the 2018 version, authors were advised against presenting an overall score and were encouraged to clearly explain how the appraisal results were interpreted and applied in the review to ensure transparency.

## Results

### Study Characteristics

**Figure 1.**
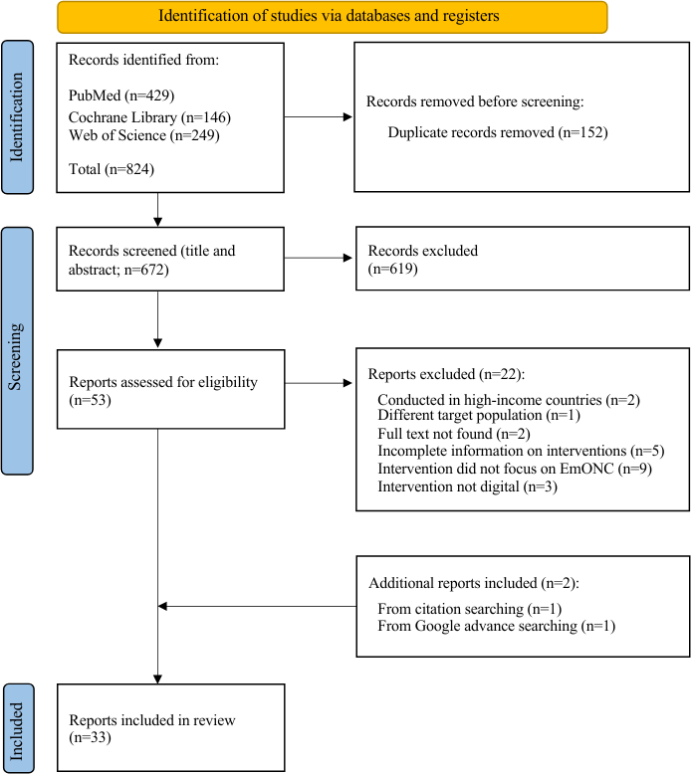
Flowchart outlining the search process for studies across databases and registries, following the PRISMA-ScR (Preferred Reporting Items for Systematic Reviews and Meta-Analyses extension for Scoping Reviews) guidelines. EmONC: emergency obstetric and newborn care.

A total of 824 studies were identified from 3 databases after the initial search, with 672 remaining after duplicate removal. After title and abstract screening, 619 studies were excluded, and the full texts of the 53 remaining studies were reviewed for eligibility. Following a critical appraisal based on the inclusion and exclusion criteria, 31 studies were selected for data extraction. Furthermore, 2 additional studies were included, one identified through citation searching and another retrieved from gray literature searching, bringing the total number of studies to 33 (Figure 1).

**Figure 2.**
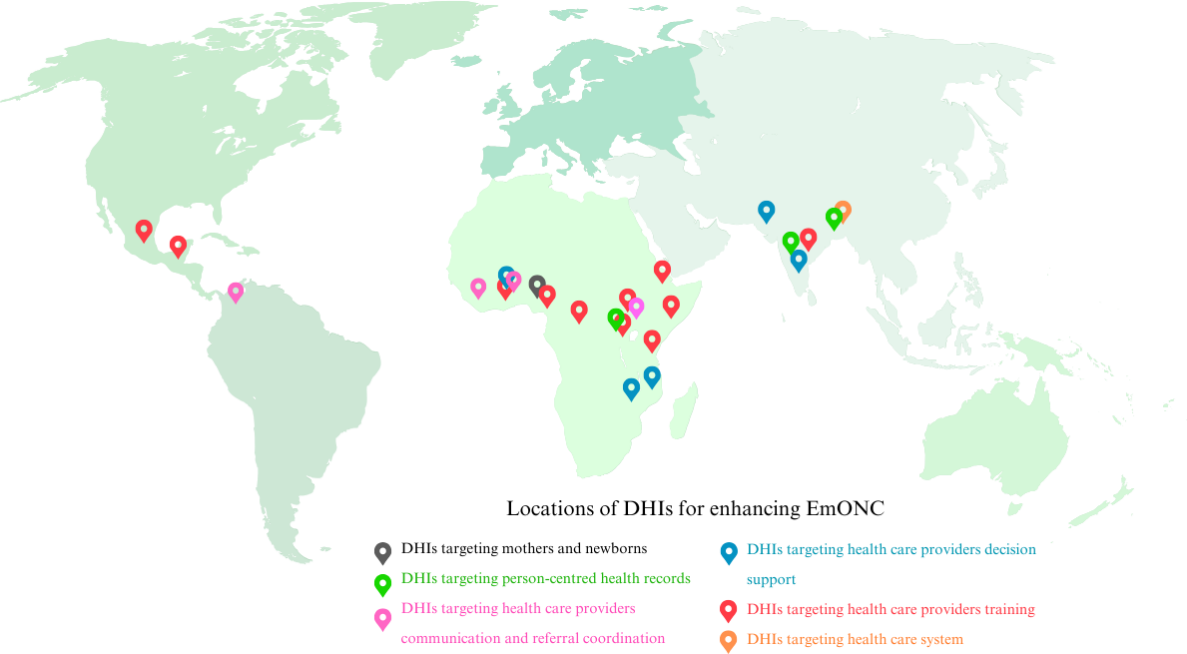
Map of DHI implementation sites for enhancing EmONC. DHI: digital health intervention; EmONC: emergency obstetric and newborn care.

These studies were conducted in 18 countries (Figure 2), with the majority of the studies taking place in India (n=5) and Ethiopia (n=4). The remaining studies were conducted in 16 LMICs across Africa (Ghana, Uganda, Liberia, Mozambique, Congo, Rwanda, Guatemala, Kenya, Tanzania, Nigeria, and Malawi), South Asia (Bangladesh and Pakistan), and the Americas (Mexico, Guatemala, and Colombia). In terms of intervention types, 21 studies addressed both obstetric and neonatal emergency care interventions, 7 studies focused exclusively on obstetric emergency care, and 5 studies focused on neonatal emergency care only. Thirteen studies reported DHIs combined with nondigital interventions, such as face-to-face mentorship or training programs, while the remaining studies exclusively used digital interventions. Tables1  2 include a detailed summary of the included studies. The study quality appraisal indicated that most of the quantitative descriptive studies had some issues with sampling, either because the sample did not match the target population or the sampling method used was inadequate (Multimedia Appendix 2).

**Table 1.** Study characteristics (N=33).

Author	Intervention name	Country	Study settings	Study population	Study design
Okonofua et al [35]	Text4Life (Centre of Excellence in Reproductive Health Innovation, University of Benin)	Nigeria	PHCs^a^ in rural areas	Pregnant women	Pre-post study
Jabeen et al [36]	Digital EmONC^b^ register	Bangladesh	District hospitals (secondary-referral services) and subdistrict hospitals (primary-referral services)	HCWs^c^, policymakers, facility managers, and statisticians	Implementation research
Gass et al [37]	Call center	India	PHCs in low-resource, geographically vast setting	Post-partum women and newborns	Prospective cohort study
Ngabo et al [38]	RapidSMS–Maternal and Child Health (UNICEF and the Rwanda Ministry of Health)	Rwanda	PHCs and hospitals in rural areas	HCWs	Pilot study
Amoakoh et al [39]	mHealth^d^ Clinical Decision-Making Support Intervention (mCDMSI)	Ghana	PHCs and hospitals	HCWs	Cluster RCT^e^
von Dadelszen et al [40]	Pre-eclampsia Integrated Estimate of Risk on the Move (PIERS-POM) app combined with Community-Level Interventions for Pre-eclampsia (CLIP; Mark Ansermino and Peter von Dadelszen)	Mozambique, Pakistan, and India	PHCs and communities	HCWs	Individual participant data meta-analysis of cluster RCT
Chiweza et al [41]	Artificial Intelligence (AI) augmented Continuous Electronic Fetal Monitoring (CEFM; Perigen Inc)	Malawi	Hospital	HCWs	Pilot study
Escobar et al [42]	Telehealth combined with an educational model (Fundación Valle del Lili [FVL])	Colombia	A tertiary high-complexity care hospital and a second-level hospital in a resource-limited region	HCWs	Descriptive ecological study
Oduro-Mensah et al [43]	Call center	Ghana	PHCs and hospitals in urban areas	HCWs	Longitudinal time series routine data analysis
Ononge et al [44]	Emergency Call and Dispatch Center (ECDC) with Kampala Digital Emergency Transport System (KDETS) mobile app	Uganda	PHCs and hospitals in urban slum communities	HCWs	Mixed method study
Lee et al [45]	Mobile Obstetric Emergency System (MORES; WhatsApp Inc)	Liberia	Rural health facilities (RHFs) and hospitals	HCWs	Pre-post study
Tuti et al [46]	The Life Saving Instructions for Emergencies (LIFE) app (Jakob Rossner)	Kenya and other low-income countries	PHCs and hospitals	HCWs and health care students	RCT
Bolan et al [47]	Safe Delivery App (SDA; Maternity Foundation and Universities of Copenhagen and Southern Denmark)	Congo	PHCs and hospitals	HCWs	Mixed method feasibility study and pilot cluster-RCT
Klokkenga et al [48]	SDA	Ghana	Hospitals in rural areas	HCWs	Cluster RCT
Lund et al [49]	SDA	Ethiopia	PHCs and hospitals in rural areas	HCWs	Cluster RCT
Thomsen et al [50]	SDA	Ethiopia	PHCs and hospitals in rural areas	HCWs	Qualitative study
Christiansen et al [51]	SDA	Ethiopia	PHCs and hospitals in rural areas	HCWs	Cluster RCT
Nishimwe et al [52]	SDA	Rwanda	Hospitals in urban and rural areas	HCWs	Pre-post study
Nishimwe et al [53]	SDA	Rwanda	Hospitals in urban and rural areas	HCWs	Pre-post study
Nishimwe et al [54]	SDA	Rwanda	Hospitals in urban and rural areas	HCWs and stakeholders	Mixed method study
Yigzaw et al [55]	Shorter, blended-learning approach (face-to-face classroom with online instruction)	Ethiopia	PHCs	HCWs	Quasi-experimental study
Walker et al [56]	PRONTO simulation and team training (PRONTO International)	Mexico	Hospitals	HCWs	Pilot study
Walker et al [57]	PRONTO simulation and team training	Guatemala	PHCs	HCWs	Pre-post study
Morgan et al [58]	PRONTO combined with Emergency Maternal and Neonatal Care Preparedness (AMANAT)	India	PHCs in urban and rural areas	HCWs	Qualitative study
Creanga et al [59]	PRONTO-AMANAT	India	PHCs in urban and rural areas	HCWs	Pre-post study
Ghosh et al [60]	PRONTO-AMANAT	India	PHCs in urban and rural areas	HCWs	Pre-post study
Miller et al [61]	PRONTO combined with The East Africa Preterm Birth Initiative (PTBi-EA)	Kenya, Uganda	PHCs and hospitals	HCWs	Pre-post study
Umoren et al [62]	electronic Helping Babies Breathe (eHBB; Rachel Umoren and Chris Paton) app and virtual reality (VR) simulations	Nigeria and Kenya	Secondary and tertiary hospitals in urban and semiurban areas	HCWs	RCT
Ndwiga et al [63]	Digital EmONC Learning and Training Assistant (DELTA) with mentoring program (Jacaranda Health and WhatsApp Inc)	Kenya	PHCs and hospitals in informal settlements	HCWs	Mixed method study
Nyamtema et al [64]	Accessing Safe Deliveries in Tanzania (ASDIT; Tanzanian Training Centre for International Health)	Tanzania	PHCs in rural areas	HCWs	Prospective cohort study
Mbaruku et al [65]	M-communication and non-pneumatic anti-shock garment (NASG)	Tanzania	PHCs and hospitals in rural areas	HCWs	Mixed method study
Akhter et al [66]	Blood Information Management Application (BIMA)	Bangladesh	A tertiary hospital and 2 proximate licensed blood banks	HCWs	Qualitative study
Rahman et al [67]	BIMA	Bangladesh	A tertiary hospital and 2 proximate licensed blood banks	HCWs	Pre-post study

a PHC: primary health care center.

b EmONC: emergency obstetric and newborn care.

c HCW: health care worker.

d mHealth: mobile health.

e RCT: randomized controlled trial.

**Table 2.** Key findings from digital health intervention studies on enhancing emergency obstetric and newborn care (EmONC; N=33).

Author	Intervention name	Barriers to DHI^a^ implementation	Study outcome
Okonofua et al [35]	Text4Life (Centre of Excellence in Reproductive Health Innovation, University of Benin)	Not reported	A small proportion of the registered women contacted the Text4Life server to request emergency transportation. Among those who made this request, most were successfully transported to primary health care facilities.
Jabeen et al [36]	Digital EmONC register	Not reported	The digital EmONC register showed strong usability and was considered highly acceptable by health care providers. The adoption, fidelity, and utility of the e-register significantly surpassed those of the traditional paper-based register.
Gass et al [37]	Call center	Not reported	The call center effectively collected postdischarge childbirth outcomes for most women participating in the study. Both accuracy and consistency of data collection were high.
Ngabo et al [38]	RapidSMS–Maternal and Child Health (UNICEF and the Rwanda Ministry of Health)	Not reported	The pilot study monitored a large number of pregnancies and recorded numerous SMS text messaging alerts for life-threatening pregnancy-related events. There was a significant rise in the proportion of deliveries that took place in health facilities, showing substantial improvement compared to the year before the pilot phase.
Amoakoh et al [39]	mHealth^c^ Clinical Decision-Making Support Intervention (mCDMSI; Mark Ansermino and Peter von Dadelszen)	Not reported	There was a positive correlation between the frequency of emergency neonatal protocol requests through mCDMSI and the total number of deliveries in the intervention clusters. This study’s findings indicate that mCDMSI did not lead to a reduction in institutional neonatal mortality.
von Dadelszen et al [40]	Pre-eclampsia Integrated Estimate of Risk on the Move (PIERS-POM) app combined with Community-Level Interventions for Pre-eclampsia (CLIP)	Not reported	Community health workers effectively conducted POM-guided visits for pregnant women in their communities, and most women accepted the recommendations made during these visits. However, the CLIP intervention did not lead to a reduction in adverse pregnancy outcomes.
Chiweza et al [41]	Artificial intelligence augmented Continuous Electronic Fetal Monitoring (CEFM; Perigen Inc)	Not reported	The pilot study observed a significant decrease in intrapartum stillbirths and early neonatal deaths, accompanied by a slight but statistically meaningful rise in the rate of cesarean deliveries.
Escobar et al [42]	Telehealth combined with an educational model (Fundación Valle del Lili [FVL])	Not reported	This intervention model reduced perinatal deaths significantly in a secondary-level hospital. Although there was a reduction in the need for blood transfusions following postpartum hemorrhage and the rate of eclampsia, a statistically significant effect on maternal outcomes was not observed.
Oduro-Mensah et al [43]	Call center	Not reported	The call center showed effectiveness in handling many calls for referral requests and securing referral beds. However, nearly half of the ambulance requests were not fulfilled due to various reasons.
Ononge et al [44]	Emergency Call and Dispatch Center (ECDC) with Kampala Digital Emergency Transport System (KDETS) mobile app (Population Services International Uganda and Kampala Capital City Authority)	Since call center agents were not health care providers, accurately interpreting health-related inquiries was challenging. Limited internet connectivity caused delays in entering data into the application. Few and inadequately equipped ambulances hindered effective implementation. Low technological skills of providers limited application uptake; some providers were reluctant to use the application.	Among all maternal and newborn health calls received by ECDC, ambulance transport was provided for the majority of the cases. Most providers reported that the ECDC and the app facilitated efficient communication, enhanced referral services, ensured equitable duty allocation, and reduced workload.
Lee et al [45]	Mobile Obstetric Emergency System (MORES; WhatsApp Inc)	Not reported	There was no statistically significant effect between the intervention’s implementation and transfer time. At the end of the study, women were more likely to have undergone a cesarean section, and newborns were less likely to be nonvigorous.
Tuti et al [46]	The Life Saving Instructions for Emergencies (LIFE) app (Jakob Rossner)	Not reported	Adaptive feedback showed a weak effect on learning gains that was not statistically significant. However, adaptive feedback demonstrated a moderate learning effect on normalized learning gains with immediate repetition, when subject-treatment interaction and differential time effects were controlled.
Bolan et al [47]	Safe Delivery App (SDA; Maternity Foundation along with the Universities of Copenhagen and Southern Denmark)	Challenging working conditions, inconsistent availability of medications and equipment, unreliable electricity supply, and inadequate salaries.	The SDA increased health care workers’ knowledge and self-confidence in managing obstetric and newborn emergencies after 3 months. Qualitative feedback supported the feasibility and acceptability of the SDA and mLearning.
Klokkenga et al [48]	SDA	Not reported	The study observed a slight but not statistically significant reduction in the incidence of postpartum hemorrhage and severe postpartum hemorrhage after the intervention.
Lund et al [49]	SDA	Not reported	The SDA significantly enhanced and maintained health care workers’ knowledge and skills in neonatal resuscitation for up to a year after its introduction. The reduction in perinatal mortality was not statistically significant.
Thomsen et al [50]	SDA	The app prompted health care providers to request medications and equipment that are typically unavailable to them. Some health care providers possess limited skills and resources, making them feel unprepared to handle complex situations. Limited access to phone charging.	Health care workers feel that the SDA has enhanced their capability to handle complications during childbirth and that they have earned greater recognition and trust within their communities. Users who attend a high number of deliveries tend to use the app more frequently during emergencies. Meanwhile, those who attend fewer deliveries mainly use the app to enhance their knowledge and to provide health education to pregnant women.
Christiansen et al [51]	SDA	Not reported	The difference in score improvements between the intervention and control groups was significant at every time point measured, indicating that the SDA more than doubled the baseline skills scores for managing postpartum hemorrhage.
Nishimwe et al [52]	SDA	Not reported	The SDA enhanced the knowledge and skills in managing postpartum hemorrhage and neonatal resuscitation, with improvements sustained up to 6 months after its introduction.
Nishimwe et al [53]	SDA	Not reported	The SDA significantly improved outcomes for both newborns and mothers following neonatal resuscitation and postpartum hemorrhage management, as observed 6 months after the baseline.
Nishimwe et al [54]	SDA	Not reported	Survey responses showed general agreement that the SDA app is easy to use and serves as an effective decision support and training tool. Nurses and midwives believed the app enhanced their ability to manage childbirth complications. Key stakeholders viewed the app as valuable, affordable, and recommended its integration into routine health care practices.
Yigzaw et al [55]	Shorter, blended-learning approach (face-to-face classroom with online instruction)	Not reported	The blended learning approach was found to be as effective as the conventional method in enhancing providers’ knowledge, but conventional learning led to better skills performance The blended learning proved more cost-effective (US $1032 vs US $1648 per trainee).
Walker et al [56]	PRONTO simulation and team training (PRONTO International)	Not reported	PRONTO received positive feedback from both trainees and nontrainees who participated in surveys and interviews, with noted improvements in their teamwork, knowledge, and self-efficacy.
Walker et al [57]	PRONTO simulation and team training	Not reported	PRONTO training was effective in enhancing providers’ knowledge and self-efficacy across all subject areas.
Morgan et al [58]	PRONTO combined with Emergency Maternal and Neonatal Care Preparedness (AMANAT; PRONTO International)	Barriers to delivering care and mentorship: physical resources, facility layout, human resources, doctor-nurse hierarchy, nurse-nurse hierarchy, corruption and fear, cultural issues, low baseline skill level, and resistance to change.	Enablers to delivering care and mentorship: enhanced skills and confidence among providers, the involvement of doctors in the training, more frequent training sessions, strong mentor-mentee relationships, administrative support, and nursing supervision and feedback
Creanga et al [59]	PRONTO-AMANAT	Not reported	After accounting for potential confounding factors, the AMANAT intervention was found significantly improving nurse-mentees’ knowledge, facility-level infection control, intrapartum care, and newborn management practices.
Ghosh et al [60]	PRONTO-AMANAT	Not reported	Results indicate a significant overall enhancement in intrapartum and newborn care practices following the AMANAT nurse-mentoring program. Facilities that conducted more simulation exercises demonstrated notably higher performance scores compared to those with fewer simulations.
Miller et al [61]	PRONTO combined with The East Africa Preterm Birth Initiative (PTBi-EA)	Not reported	Knowledge scores showed significant improvements, particularly in maternal care, neonatal care, and communication techniques. The proportion of preterm birth-related evidence-based practices accurately performed during simulations also increased significantly, with progress observed in maternal, neonatal, and communication-related areas.
Umoren et al [62]	electronic Helping Babies Breathe (eHBB; Rachel Umoren and Chris Paton) app and virtual reality (VR) simulations	Not reported	Performance at 6 months postintervention was similar among all groups, but the decline in bag-and-mask ventilation skills over time was significant in the video and control groups but not in the VR group. In the VR group, pass rates for OSCE^b^ B were higher at three and six months than immediately after the intervention.
Ndwiga et al [63]	Digital EmONC Learning and Training Assistant (DELTA) with mentoring program (Jacaranda Health and WhatsApp Inc)	Power dynamics between different staff levels and inconsistent attendance posed challenges. Reluctance to change the existing approach to care management in the hospital, particularly from senior health care providers.	The results showed significant improvement in the ability to recognize and manage various obstetric conditions, including antepartum and postpartum hemorrhage, retained placenta, obstructed labor, pre-eclampsia or eclampsia, and puerperal sepsis. Significant improvements were observed across both public and private health facilities.
Nyamtema et al [64]	Accessing Safe Deliveries in Tanzania (ASDIT; Tanzanian Training Centre for International Health [TTCIH])	Not reported	Training, virtual teleconsultation, on-site supportive supervision, and continuous mentorship increased the proportion of women receiving emergency obstetric care significantly from the baseline period to the intervention phase. The direct obstetric case fatality rate showed a slight decrease in both the intervention and control groups over time.
Mbaruku et al [65]	M-communication and non-pneumatic anti-shock garment (NASG)	Ambulances or other transportation were requested for referrals but failed to arrive. Some facilities were unable to send reports due to low network coverage or other technical obstacles.	The use of a Closed User Group to support the implementation of the NASG showed high acceptance and usability in treating hypovolemic shock caused by obstetric hemorrhage. The case fatality rate significantly declined during the project.
Akhter et al [66]	Blood Information Management Application (BIMA)	High turnover of medical staff. Doctors in the obstetric ward relied on unlicensed blood brokers for emergency blood supplies. Limited on-site blood availability.	Patients’ attendants were often clueless about blood availability in emergencies, so the most common way to obtain urgent blood was through unlicensed blood brokers. Both health care providers and patients’ attendants relied heavily on a network of unlicensed blood brokers to secure blood for emergency postpartum hemorrhage patients, despite the inability to verify the quality or type of blood provided. Unlicensed blood brokers believed they were offering a necessary service, motivated by significant financial gain, but were unaware of the safety concerns regarding the blood they supplied. They also disclosed having strong networks and unethical connections with professional blood donors and blood banks.
Rahman et al [67]	BIMA	Not reported	Following the BIMA intervention, the median time from identifying the need for blood to receiving a blood transfusion was significantly reduced, even after adjusting for factors such as maternal age, education, parity, provider shifts, and reasons for transfusion.

a DHI: digital health intervention.

b OSCE: objective structured clinical examination.

c mHealth: mobile health.

**Figure 3.**
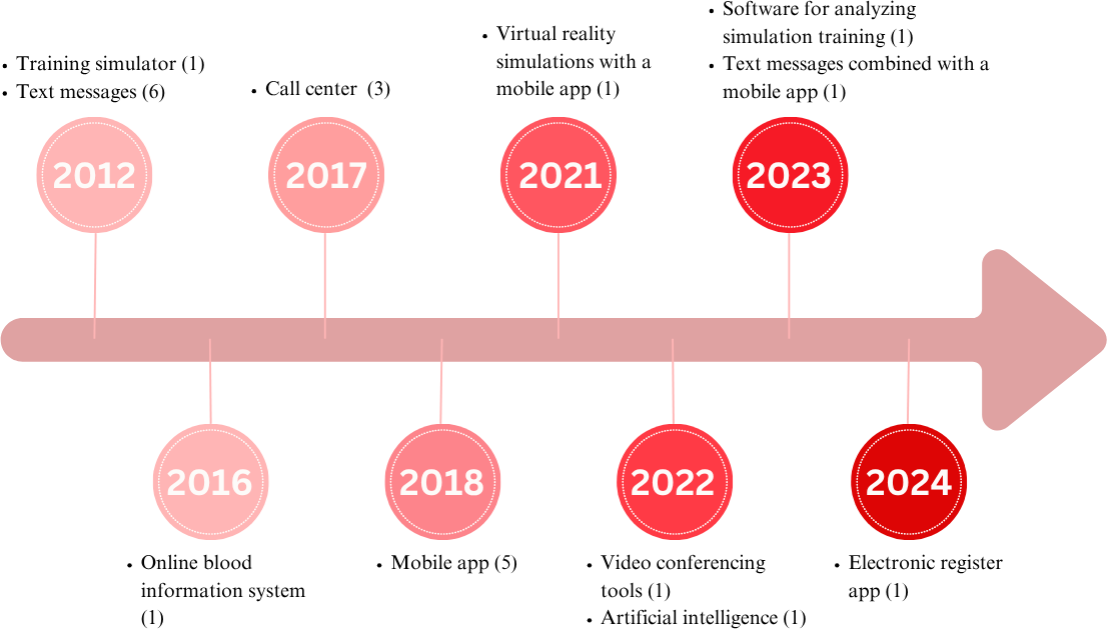
The mode of delivery for digital interventions used to improve emergency obstetric and newborn care (EmONC) services over the years (by publication year). The E-register app refers to the electronic registration of health care records.

A total of 21 unique DHIs were reported in these studies. Multimedia Appendix 3 summarizes the purpose, features, and functionalities of these DHIs, along with their WHO CDISAH classification [12]. Most DHIs targeted health care providers (n=19), while one intervention targeted pregnant women and another intervention targeted health care systems. The earliest DHI identified in this review was developed in 2012, which applied a training simulator for simulation-based learning of health care providers and a SMS text messaging for emergency alerts. In the following years, other interventions, such as online blood information system and e-register app, were also used in this field (Figure 3).

**Figure 4.**
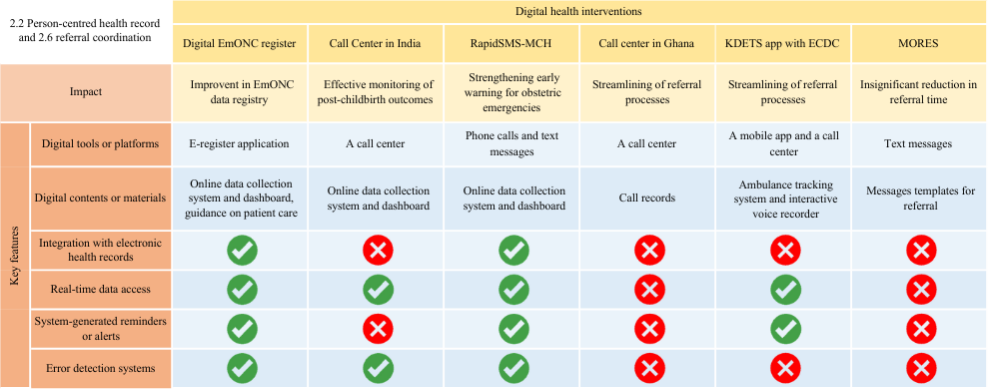
Key features of digital health interventions (DHIs) for person-centered health records and referral coordination and their associated outcomes. ECDC: Emergency Call and Dispatch Center; EmONC: Emergency Obstetric and Newborn Care; KDETS: Kampala Digital Emergency Transport System; MORES: Mobile Obstetric Emergency System; RapidSMS-MCH: RapidSMS–Maternal and Child Health.

The connections between essential DHI features and their associated outcomes are demonstrated in Figures4^6^, where cross marks indicate features that are unavailable and question marks indicate features that were not described in adequate detail and are therefore unclear.

**Figure 5.**
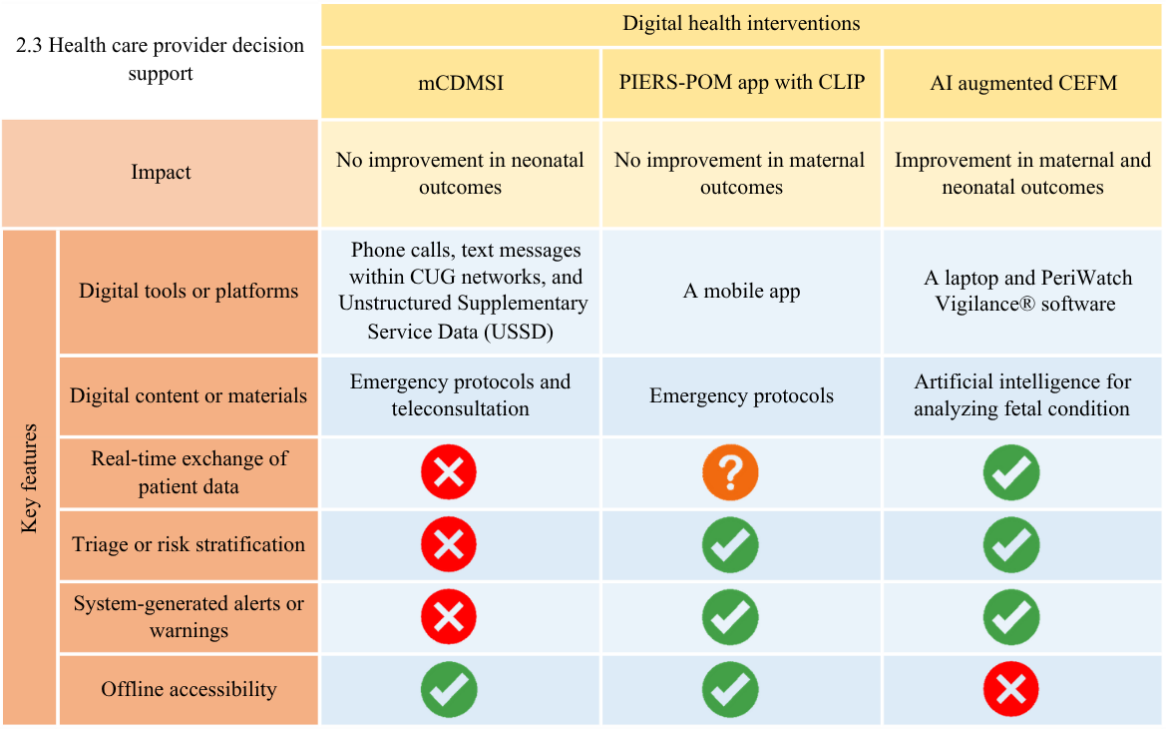
Key features of digital health interventions (DHIs) for health care provider decision support and their associated outcomes. AI: artificial intelligence; CEFM: Continuous Electronic Fetal Monitoring; CLIP: Community-Level Interventions for Preeclampsia; CUG: Closed User Group; mCDMSI: mHealth Clinical Decision-Making Support Intervention; PIERS-POM: Pre-eclampsia Integrated Estimate of Risk on the Move.

**Figure 6.**
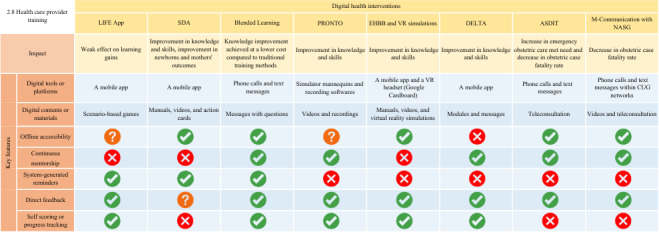
Key features of digital health interventions (DHIs) for health care provider training. Check marks indicate the presence of features and their associated outcomes. ASDIT: Accessing Safe Deliveries in Tanzania; CUG: Closed User Group; DELTA: Digital EmONC Learning and Training Assistant; EHBB: electronic Helping Babies Breathe; LIFE: Life Saving Instructions for Emergencies; NASG: Nonpneumatic Anti-shock Garment; PRONTO: Programa de Rescate Obstétrico y Neonatal: el Tratamiento Óptimo y Oportuno; SDA: Safe Delivery App; VR: virtual reality.

### DHIs Targeting Pregnant Women

Text4Life, implemented in Nigeria, was the only intervention targeted at pregnant women [35]. This DHI allowed participating users (n=1620) to report emergencies by text message and get transported to nearby health facilities. Text4Life facilitated an alert messaging system between patients and health care providers, as well as included the functionalities for tracking and viewing patient data, reports, and other administrative details. In total, 56 women used the intervention to request emergency transportation during the study period. Among them, 51 (91.1%) women were transported to primary health care facilities on time, 4 (7.1%) women could not be transported due to the unavailability of taxis, and 1 (1.8%) woman gave birth at home before the taxi could arrive.

### DHIs Targeting Health Care Providers

#### Person-Centered Health Records

Three studies examined the implementation of digitized records for capturing, storing, accessing, and sharing the health information of mothers and newborns [36-38]. While data collection methods varied in these studies, digitized patient data were used for monitoring in all cases using web-based dashboards accessible to frontline health care providers via mobile devices. The first study conducted in Rwanda implemented the RapidSMS-Maternal and Child Health (MCH; UNICEF and Rwanda Ministry of Health) system that allowed CHWs to register new pregnancies in their respective areas to provide effective monitoring throughout pregnancy and postdelivery. About 81% (n=11,502) of the district’s estimated annual pregnancies were recorded during its 1-year implementation period, with CHWs achieving a 100% reporting compliance rate [38]. The second study was a cohort study conducted in India that used a call center to monitor the health conditions of mothers and newborns post-discharge. The center effectively monitored the health of mothers and newborns through 157,689 calls using their questionnaire (98% accuracy and 93.7% validity), with a high follow-up rate of 99.7% [37]. The third study introduced an e-register to record inpatient data in selected EmONC facilities in Bangladesh. Health care providers reported high satisfaction levels of usability and acceptability with the e-register, which also demonstrated significantly better adoption, fidelity, and utility compared to the traditional paper-based register [36]. These studies sought not just to digitize health records but also to enhance the quality, timeliness, and completeness of patient data, recognizing that in time-critical emergencies, having accurate and readily accessible information can significantly influence clinical decisions and outcomes.

#### Health Care Provider Decision Support

Three studies focused on DHIs providing support for health care providers at the point of care: Pre-eclampsia Integrated Estimate of Risk on the Move (PIERS-POM; Mark Ansermino and Peter von Dadelszen) application to identify and manage hypertensive pregnancies during home visits [40]; Unstructured Supplementary Service Data (USSD) to provide neonatal emergency protocols [39]; and use of artificial intelligence (AI) to augment fetal monitoring [41]. First, a meta-analysis conducted by Dadelszen et al [40] used the PIERS-POM app designed to assist health care providers with triage and recommend the initial treatment of severe hypertension and suspected severe pre-eclampsia. No significant difference in adverse pregnancy outcomes was observed between the PIERS-POM app intervention and control clusters across 3 countries (n=69,330), although there was high acceptability among women who received care through PIERS-POM.

Second, Amoakoh et al [39] used USSD to provide neonatal emergency protocols. USSD is an interactive communication protocol that enables health care providers to request specific guidelines related to neonatal emergencies. It can be accessed at no cost using Closed User Group (CUG) SIM cards, which also enable free calls within the network. This system is particularly valuable in low-resource settings, where internet access is limited and offers critical support during emergencies. Throughout the intervention period, health care providers made 5329 requests to USSD. A strong positive correlation of 0.71 (*P*=.05) was observed between the number of USSD requests and the total number of deliveries within the intervention clusters. Although this intervention was found to be well-used by health care providers, it did not reduce institutional neonatal mortality [39].

The last study integrated AI into Continuous Electronic Fetal Monitoring [41]. This AI-enhanced Continuous Electronic Fetal Monitoring system used a risk stratification algorithm to detect irregularities in fetal heart rate and labor progression. Alerts were triggered in case of any irregularities, and the cases were escalated to consultant-level physicians. Preliminary data comparing outcomes from the 6 months pre- and post-implementation (n=7926) revealed a significant reduction in intrapartum stillbirths (0.37%; *P<*.001) and early neonatal deaths (0.58%, *P*<.001), alongside a rise in cesarean section rates (2.1%; *P*=.004) [40]. Nonetheless, these early findings are susceptible to selection and expectancy bias, which limits the strength of the conclusions.

#### Health Care Provider Communication and Referral Coordination

Four studies assessed DHIs for communicating information among health care providers and health care system managers. A study conducted in Colombia [42] used a telehealth and education model to manage obstetric and neonatal emergencies for 250 patients, facilitating effective communication and cooperation between a secondary hospital and a tertiary hospital. The clinical indicators from the study demonstrated that this intervention significantly lowered the odds of perinatal mortality by 78% (adjusted odds ratio 0.22, 95% CI 0.07-0.71; *P*=.02).

Three studies conducted in Ghana [43], Uganda [44], and Liberia [45] focused on improving referral systems. A call center intervention in Ghana allowed frontline health care providers to request EmONC referrals and efficiently process 346 referral cases [43]. Similarly, a larger study in Uganda combined a call center with an ambulance mobile app and received 2911 maternal and neonatal referral calls [44]. In Uganda, ambulances were dispatched for 84% (n=2446) of the EmONC calls, whereas 61.2% (n=85) of the ambulance calls were addressed in Ghana. The study conducted in Liberia used the text messaging application WhatsApp (Jan Koum and Brian Acton) as a platform to improve the referral process between facilities. The intervention resulted in an increased number of emergency cesarean sections carried out in referred facilities and a decrease in the number of nonvigorous newborns at birth, although transfer time between facilities did not improve significantly [45].

#### Health Care Provider Training

A total of 20 studies used 7 DHIs to deliver EmONC educational and training content to health care providers. Among these, 6 were mobile apps: Safe Delivery App (SDA; n=8; Maternity Foundation, together with the Universities of Copenhagen and Southern Denmark), PRONTO simulation and team training (n=6; PRONTO International), Life-Saving Instruction for Emergencies (n=1; Jakob Rossner) app, Digital EmONC Learning and Training Assistant (DELTA; n=1; Jacaranda Health and WhatsApp Inc), electronic Helping Babies Breathe (eHBB; Rachel Umoren and Chris Paton) app and virtual reality (VR; n=1) simulations, and Accessing Safe Deliveries in Tanzania (ASDIT; n=1; Tanzanian Training Centre for International Health [TTCIH]). There were also 2 studies that used text messaging and phone calls as the digital intervention.

The SDA used animated videos to describe EmONC management and was most widely implemented, with studies conducted in Congo, Ghana, Ethiopia, and Rwanda [47-54]. The study enrolled a total of 455 health care providers, along with 6 hospital managers and directors, who collectively provided care for 3601 women. Studies demonstrated high feasibility and acceptability among health care providers [47,50,54], as well as significant improvements in health care providers’ knowledge and skills related to postpartum hemorrhage and neonatal resuscitation [47,49,51,52]. However, mixed results were reported on other outcomes, including the incidence and outcomes of postpartum hemorrhage and perinatal outcomes following the implementation of SDA [48,49,53]. Similar to SDA, the Life-Saving Instruction for Emergencies app implemented in Kenya provided individual training and adaptive feedback focused on emergency neonatal care. The adaptive feedback was designed to prompt health care providers to reflect on incorrect care decisions made during training. Their findings suggested greater learning gains using adaptive feedback compared to standardized feedback [46].

Two other studies also enhanced individual learning through simpler methods, such as text messaging and phone calls. Yigzaw et al [55] aimed to reduce the duration of the traditional onsite EmONC training program in Ethiopia by using automated and targeted SMS text messaging and phone calls to extend individual learning. The study found that this approach was as effective as the conventional method in improving EmONC knowledge and demonstrated greater cost-effectiveness, costing US $1032 per trainee compared to US $1648 with the traditional method [55]. Mbaruku et al [65] used a CUG network to implement training videos of the non-pneumatic anti-shock garment module for managing obstetric bleeding. The study demonstrated a high level of acceptability and usage of the CUG-nonpneumatic anti-shock garment, as it was used in 70.8% (n=297) of women suspected of having obstetric hemorrhage during the intervention [65].

The PRONTO program was implemented in 5 countries, making it the most widely used simulation-based team learning and training initiative. PRONTO used videos, PartoPants mannequins for vaginal delivery, and NeoNatalie infant mannequins in their training. The training sessions were recorded and analyzed with software to assess the simulation practice. It was implemented as a sole intervention in Mexico and Guatemala [56,57] and was combined with mentoring programs in India [58-60] and Kenya and Uganda [61]. Although the implementation period varied across studies, all reported improvements in EmONC clinical knowledge and practice following the scenario-based simulations.

Similarly, the DELTA [63], ASDIT [64], and eHBB-VR [62] interventions combined DHIs with mentoring programs. First, the DELTA intervention is a self-directed digital learning platform that enabled nurse-mentors to input data and monitor progress across target facilities in real-time. The study found notable improvements in EmONC knowledge at all health care levels, including better detection and management of obstetric complications [63]. Second, the ASDIT intervention used virtual teleconsultation following onsite training sessions and continued their mentorship through communication platforms such as telephone and social media. Although the study did not evaluate the providers’ knowledge and skills postintervention, it reported a slight reduction in the direct obstetric case fatality rate [64].

The final study, the eHBB-VR intervention, assessed the effectiveness of VR combined with a digital guide in retaining neonatal resuscitation skills [62]. The study evaluated bag-and-mask ventilation skills, as well as performance on objective structured clinical examinations A and B, the latter involving prolonged neonatal resuscitation scenarios. At 6 months, nurses and midwives in Kenya and Nigeria showed similar overall pass rates in neonatal resuscitation skills across all groups. However, post hoc analysis revealed that the VR group performed significantly better than the control group on specific objective structured clinical examination B assessments, including opening the mouth slightly, squeezing the bag harder, and achieving the target ventilation rate. Participant feedback for both the VR and video refresher training interventions was generally positive [62].

### DHIs Targeting Health Management Systems

Two studies described a DHI targeting supply chain management systems in Bangladesh, called the Blood Information Management Application (BIMA) [66,67]. As an online application for managing blood bank information, BIMA offered access to 2 blood bank databases within and outside the hospital. A pre-post study involving 310 women with obstetric bleeding demonstrated that BIMA significantly reduced the median time from identifying the need for blood to the actual transfusion by 24 minutes (95% CI −37.61 to −12.39; *P*<.001) [67]. For women with pregnancy-induced hypertension, the transfusion time was reduced by 123 minutes, and for women with postpartum hemorrhage, it was reduced by 62.5 minutes. A qualitative study agreed that BIMA could simplify the blood transfusion process, making it more affordable and accessible anytime for patients [66]. However, the intervention’s effectiveness was significantly compromised as both service providers and recipients depended heavily on an unregulated group of unlicensed blood brokers in Bangladesh.

### Barriers to DHI Implementation

Challenges related to DHI implementation were documented in 7 studies [44,47,50,58,63,65,66]. Several barriers to successful implementation were identified, with key themes including health care system preparedness, workforce limitations, inadequate digital infrastructure, and challenges related to cultural norms or personal beliefs.

Health care system preparedness was the most common barrier mentioned in the selected studies. Three studies reported a shortage of medications and medical equipment necessary for training staff or practicing EmONC [47,50,58]. Given that such resources are essential for delivering optimal emergency care, the lack of these may have impeded the successful implementation of DHIs and hindered optimal outcomes. In the case of BIMA, which managed blood bank information, the limited blood supply in the blood bank hindered the establishment of a digitalized blood bank system [66]. Two studies explored the use of digital technologies to improve EmONC’s ambulance service efficiency, highlighting the shortage of ambulances as a key challenge in the referral system [44,65]. The lack of adequate first-aid kits in the available ambulances further exacerbated the issue [44].

In addition to insufficient medications and equipment, workforce limitations were identified as a barrier in the health care systems across the 7 studies. Even when DHIs were introduced to provide EmONC training and improve health care providers’ skills, the interventions alone were not adequate to improve health care providers’ attitude toward adoption and confidence in delivering care due to the shortage of health care resources in resource-limited settings [50,65]. In addition, the availability of skilled health care providers was minimal, largely due to high staff turnover and inability to retain trained and skilled individuals [63,66]. This issue was compounded by some health care providers’ low practical and technological skills [44,58]. Poor salaries of health care providers in LMICs could also be disincentivizing [47].

Inadequate technical and digital infrastructure can restrict the implementation of DHIs and was reported as a barrier in 3 studies [44,50,65]. For example, stable internet connectivity is vital during emergencies to handle data promptly [44,65]. In addition, Thomsen et al [50] noted that health care providers often faced difficulties charging their phone batteries for using the SDA at health care facilities, posing an additional challenge in implementing DHIs in remote regions.

Finally, DHIs also face challenges related to cultural norms or personal beliefs. Morgan et al [58] identified several factors that could impede EmONC services, including restricting male doctors from some delivery rooms and a stronger family preference to care for male newborns. Some health care providers also exhibited resistance to change in practice [58,63], though no additional details were provided in these studies.

**Figure 7.**
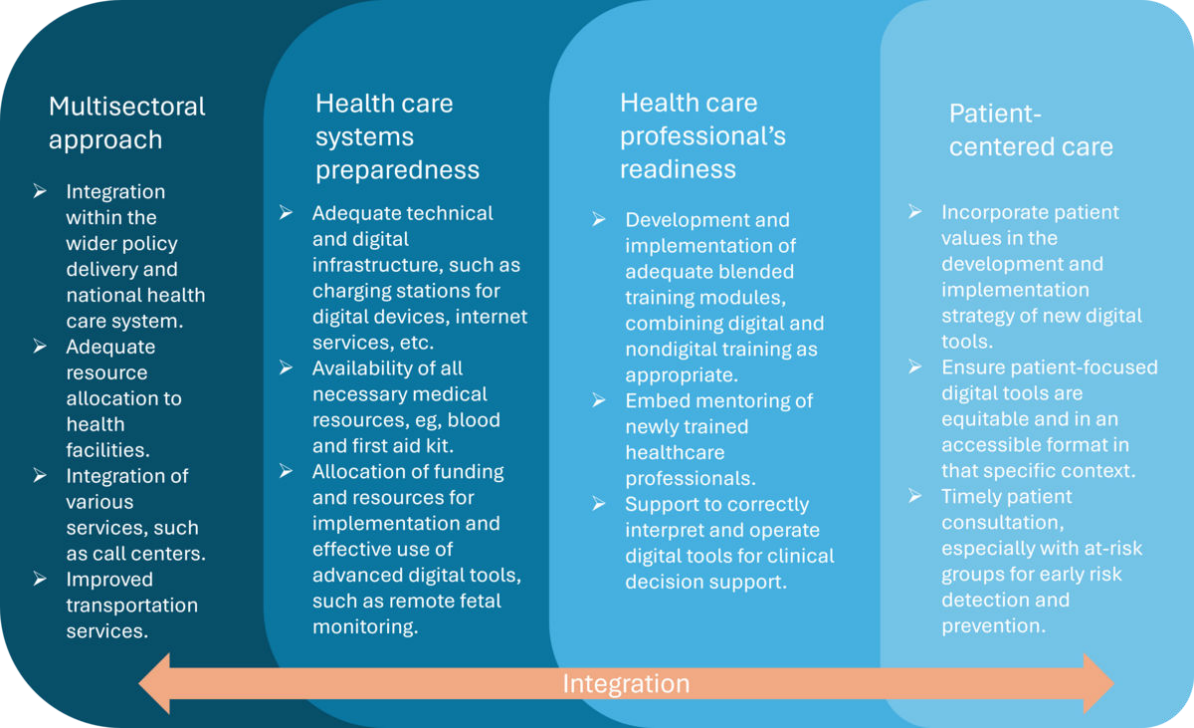
Framework for digital intervention development and implementation for emergency obstetric and newborn care.

Based on our synthesis of the DHIs developed and implemented in LMICs, we have developed a framework that outlines the key considerations that need to be in place across 4 domains (Figure 7): (1) multisectoral approach, (2) health care systems preparedness, (3) health care professionals’ readiness, and (4) patient-centered care. These domains are interlinked in a dynamic manner and should be integrated for effective development and delivery of contextualized digital interventions for EmONC. Due to the nature of EmONC services, a multisectoral approach is necessary to ensure that DHIs are not stand-alone but integrated within the national health services and broader health care systems. This is also important for scaling up such interventions in the future. Health facilities should be equipped with all relevant resources and associated services for seamless and timely delivery, such as call centers and transportation services. Health care systems should be provided with the necessary technical infrastructure, essential medical resources, and adequate funding for the implementation and effective use of digital health tools. It is also important to support health care professionals with the right level and mode of training to ensure that they are confident in delivering care using a range of DHIs, especially in a highly stressful emergency care setting. Finally, mothers’ and their informal caregivers’ values should be incorporated into the development and implementation of such digital interventions. Improved patient-centered care can help with early detection and prevention and save time and resources in the long term.

## Discussion

### Principal Findings

#### Overview

This scoping review consolidated comprehensive evidence from published literature to date on DHIs to enhance access to and the quality of EmONC for pregnant women and newborns in LMICs. While the purposes, use cases, and modes of delivery of these digital interventions varied, 4 general themes emerged across their implementation: stagnant progress of DHI development and implementation for EmONC services in LMICs; the most common purpose of implemented DHIs is education and training delivery; health care providers are the main target users for such DHIs; and diversity in the applicability of DHIs across different health care settings (from primary health care services to tertiary hospitals) to improve EmONC access.

#### Limited Progress in the Development and Implementation

Evidence synthesis demonstrated slow progress in the development of DHIs for EmONC services suitable for low-resource settings. The most prevalent mode of delivery in these included studies was mHealth, which included 6 mobile apps, 6 text messaging features (SMS text messaging and USSD), and 1 intervention combining text messaging with a mobile app. However, from a global perspective, more advanced mHealth interventions targeting maternal and neonatal care have been implemented in high-income countries than in LMICs [19,68]. While emerging technologies such as VR [62] and AI [41] have started to be introduced in LMICs, their adoption remains limited due to barriers identified in this review. For example, a basic cardboard VR headset was used in Nigeria and Kenya [62], whereas studies conducted in high-income countries have used more immersive VR technologies in training health care workers, such as head-mounted displays or Cave Automatic Virtual Environments [21]. Other examples of advanced and effective technologies widely used in higher-resourced emergency care settings include video games and augmented reality [69,70].

The contrast in adopting digital technologies between these 2 settings might be attributed to several context-specific barriers that hinder the innovation and implementation of DHIs in LMICs. In settings where critical resources—such as medications, medical equipment, skilled health care providers, and ambulances for emergency referrals—are scarce, implementing complex digital solutions such as AI-assisted systems, which demand advanced technical infrastructure and training, may not yield sustainable outcomes. In resource-limited settings, introducing DHIs can increase oversight of performance and efficiency, potentially revealing system and institutional dysfunctions [71]. For example, the adoption of advanced technologies such as AI in LMICs is likely to highlight and draw greater attention to challenges such as unreliable electricity, weak telecommunications networks, limited mobile access, and insufficient educational infrastructure [72]. Addressing these particular gaps is essential to enable equitable involvement in developing, researching, and regulating such digital technologies in LMICs.

#### DHIs Are Mostly Intended for Educational and Training Purposes

The second theme of DHIs examined in this field was to deliver education and training materials digitally to health care providers, as reported across 33 studies and 21 distinct DHIs. These training-focused DHIs addressed EmONC signal functions within their materials, and the interventions primarily involved mobile apps and telemedicine. The findings of this review showed generally positive outcomes in health care providers’ clinical practices, consistent with several systematic reviews and meta-analyses in this field [21,25,73]. This study’s findings also suggest that DHIs can enhance access to EmONC by streamlining the referral system, providing affordable or free mobile communication between patients and health care providers, supporting data collection, and providing integrated diagnostic and clinical decision support. Due to challenges faced by health care systems in resource-limited settings, most of the training materials were adapted to the local context to enhance students’ understanding and relevance of the content. Training materials can also promote low-tech, low-cost solutions that enhance care delivery, such as using the Augmented Infant Resuscitator to address neonatal hypoxia [74].

Despite progress in digitizing health care provider training, many aspects of EmONC services remain unaddressed. The WHO CDISAH could serve as a practical guide for selecting appropriate DHIs for other specific use cases in this field [12]. This study identified just 7 out of the 32 categories outlined in the WHO CDISAH, indicating the need to focus more on other potential types of digital interventions. For example, issues related to the inventory of drugs used in EmONC signal functions could be addressed by examining the “3.2 Supply Chain Management” category in WHO CDISAH [12]. Interventions may include using communication systems and data dashboards to monitor and report on drug supply and usage. A systematic review by Agarwal et al [75] also highlighted the need for further research to compare mobile device–based stock notifications and commodity management systems with traditional paper-based methods. Implementing a wider range of DHIs beyond the identified 7 categories could lead to a more comprehensive improvement in EmONC services, especially in resource-limited settings.

#### The Interventions Are Mainly Aimed at Health Care Providers

The third theme highlights that health care providers are targeted as the end-users for the majority of DHIs. Of 33 selected studies, only the Text4Life app and a call center targeted pregnant women [35,37]. It suggests a significant gap in the use of DHIs among pregnant women and mothers, who could greatly benefit from these technologies. Building on the example of the Text4Life app [35], which enables mother-led reporting, DHIs can also be leveraged to create peer support groups among at-risk expectant mothers and families. For instance, DHIs can facilitate communication within peer support groups, allowing at-risk mothers and their families to exchange practical advice, prepare for emergencies, and receive emotional support, which can be especially valuable in remote or underserved areas. Similarly, Feroz et al [23] noticed the gap and recommended developing more digital tools specifically designed for pregnant women as the primary users in pre-eclampsia and eclampsia interventions to encourage personal health monitoring.

Low levels of digital literacy are usually a major challenge when targeting patients as DHIs users in LMICs, affecting individuals’ ability and confidence in using digital technologies [76,77]. Moreover, digital interventions designed solely for use during medical emergencies may not have a long-term use case for expecting parents and caregivers. Hence, a continuum of care approach is more appropriate, which encompasses all stages of pregnancy care. As a result, digital interventions for expectant mothers often aim to prevent obstetric and neonatal complications, such as by improving antenatal care coverage [17], enhancing nutritional intake during pregnancy [78], or supporting a healthy prepregnancy lifestyle [79]. While such interventions are valuable, it is also important to incorporate features usable during emergency events. Simplified features, such as an emergency button with integrated location sharing or a voice-assisted symptom checker, may improve usability and accessibility for mothers in emergencies. It is also essential that DHIs offer offline capabilities for mothers residing in areas without internet connectivity.

Furthermore, this study acknowledged that most of the included studies lacked an impact evaluation of maternal and perinatal health outcomes to assess the effectiveness of the intervention. In the studies that included such evaluations, the outcomes varied depending on the type of intervention. Similar findings were reported in another review, despite most studies being conducted in high-resource settings [21]. Maternal and perinatal outcomes may be overlooked in these studies, as most DHIs focus on health care providers, aiming to enhance their knowledge and skills. The issue may be more evident in LMICs due to their differing capacities for collecting data on maternal and perinatal outcomes, making direct comparison and analysis more challenging [45,48]. Hence, aside from WHO’s guidelines on measuring maternal mortality [80], there remains a need for global guidelines to standardize the measurement of other maternal and perinatal health outcomes following interventions.

#### DHI Implementation Across Different Health Care Settings

Finally, some studies demonstrated better integration across settings, from primary health care centers offering essential EmONC services to tertiary hospitals equipped to provide comprehensive EmONC services. For example, Escobar et al [42] achieved a reduction in perinatal mortality by implementing a telemedicine system that connected a tertiary high-complexity care hospital with a second-level hospital in a resource-limited region. This supports the concept that EmONC represents a continuum of services across functioning health facilities, and providing quality care must be accessible to all [81]. Moreover, the included studies were concentrated in Africa and South Asia, regions with the highest rates of maternal and neonatal deaths, making these insights more relevant [82]. Given the identified benefits of DHIs identified in this study, this evidence synthesis may provide valuable insights for future innovations designed to reduce the global burden of maternal and neonatal deaths through digital advancements in EmONC services.

### Future Trajectory of Digital EmONC

The findings from this review highlight the range of digital innovations that have been implemented to provide emergency care to mothers and newborns. To provide personalized care tailored to specific conditions and risk factors, a multifaceted approach is necessary, as digital tools that are not integrated into the health care systems are often not deemed sustainable and interoperable in the long term. This approach includes: (1) maintaining updated digital health records for routine check-up, monitoring, and follow-up [36-38] as well as connecting other services, such as ambulance and call centers with the patients [44]; (2) implementation of emerging digital tools, such as AI-powered decision support systems [41] to help with early detection of risks; and (3) context-specific, blended learning models for health care professionals that will enable capacity building in low-resource settings and effective use of such digital tools [46-65].

For instance, our analysis indicated that the integration of features such as real-time patient data exchange is crucial for interventions designed to support health care providers in EmONC decision-making (Figure 5). Nevertheless, as this feature is highly dependent on stable internet connectivity (such as when using AI), future interventions should prioritize the development of reliable internet infrastructure within the target areas. Another example is that “direct feedback” is regarded as an essential feature in DHIs aimed at enhancing health care provider learning (Figure 6). Therefore, DHIs that can deliver timely and relevant information to learners about their performance will facilitate their understanding of their current knowledge and foster active learning better.

Moreover, to effectively improve EmONC in low-resource settings, it is crucial to shift the focus of DHIs from merely introducing digital tools for care delivery to addressing fundamental health care system challenges, such as using DHIs to assess and distribute EmONC-related resources more equitably. This provides a more structured approach for researchers and program planners to use the most suitable DHIs that can be seamlessly integrated into the EmONC framework [5,7]. Our proposed framework (Figure 7) reflects these findings and can be used when developing and implementing digital tools for low-resource settings in the future. In addition, future research can investigate the potential of DHIs to enable real-time tracking of medication stock levels, assist in forecasting demand for certain drugs, or help health care workers manage and coordinate shifts and workloads in these settings.

As recommended by the non-adoption, abandonment, scale-up, spread, and sustainability framework [83], development and implementation of future integrated digital health technologies for EmONC should be context-specific, taking into consideration various stakeholder needs, resource availability, value proposition, and how this system fits within the wider system. Active involvement of all user groups in the co-creation of such digital interventions and implementation strategies through an agile, user-centered approach will ensure integration within the existing health care model and long-term use.

### Recommendations and Implications for Future Research

Many system-level challenges affecting the implementation of DHIs often extend beyond the scope of a single project. To address this, future DHI initiatives should be thoughtfully co-designed with local stakeholders to ensure that these are accessible and acceptable to health care providers in remote areas while also being manageable and sustainable with minimal resources. Establishing strong collaboration among stakeholders should begin early in the planning phases and continue through to full-scale deployment, with the goal of co-creating context-specific DHIs that integrate multisectoral strategies [71,84]. The WHO-International Telecommunications Union toolkit for national eHealth strategies offers a comprehensive framework to guide pilot projects, scale-up efforts, or strategy updates, highlighting key conditions such as supportive policies, a skilled workforce, and robust technological infrastructure [85]. Addressing digital infrastructure and technical capacity in low-resource settings is also crucial, as these factors pose significant barriers. Further research is necessary to determine the best ways to incorporate cultural and literacy considerations into the design of digital technology for EmONC services, especially in LMICs.

The findings of this review also emphasize the need for more rigorous, controlled studies to evaluate the effectiveness of DHIs for improving maternal and perinatal health outcomes in LMICs and, therefore, could help raise awareness among researchers, clinicians, and policymakers to study various DHI modalities in such settings.

Finally, the cost-effectiveness of these interventions must be assessed to support the scale-up of high-value DHIs in this field. Several frameworks exist to guide the planning, execution, and interpretation of economic evaluations for DHIs, including the World Bank’s “Framework for the Economic Evaluation of Digital Health Interventions” [86,87], which is specifically designed for use in resource-limited countries. Despite existing research gaps, the increasing availability of digital technologies holds significant potential for improving access to and the quality of EmONC services.

### Limitations

While this scoping review incorporates both peer-reviewed publications and gray literature, a key limitation is the lack of gray literature identified. Given the wide spectrum of digital interventions, it is possible that some unpublished initiatives or pilot projects were not captured. Study designs varied across studies due to the heterogeneity of intervention types and outcome measures of the selected studies. While RCTs are considered the gold standard in clinical studies, implementation-based studies often adopt quasi-experimental methods for pragmatic reasons, such as pre-post designs with limited bias control. Several studies also had small sample sizes [46,47,56], which can limit the power of the findings and the ability to draw meaningful conclusions. There was also variability in study quality in terms of the appropriate reporting of findings. While the studies included in this scoping review provide useful insights into the gradual adoption and proliferation of digital health in EmONC, more robust evaluation of different types of interventions is needed in the future, which could be assessed using meta-analysis methods.

### Conclusion

The findings from this scoping review highlight the diverse yet limited advancements in digital interventions to enhance access to and the quality of EmONC in LMICs. Despite the identified benefits of DHIs, such as improved clinical practices and referral systems among health care providers, there remains a significant lag in innovation. This is often due to a lack of essential equipment, adequate human resources, and technical infrastructures. Most interventions targeted health care providers rather than patients, due to challenges related to digital literacy and the complexity of managing emergencies in low-resource settings. This review also highlights a significant gap in research regarding the management of human resources and supply chains in EmONC, emphasizing the need for DHIs to address these critical areas. Future research should focus on developing accessible and sustainable DHIs that support a continuum of EmONC services, especially in low-resource settings.

Given the identified benefits of DHIs in this study, this evidence synthesis may provide valuable insights for further future innovations aimed at supporting progress toward achieving Sustainable Development Goals 3.1 and 3.2, which focus on reducing global maternal and neonatal mortality. By positioning this synthesis as both a scholarly contribution and a policy-relevant resource, it offers actionable guidance to health care stakeholders, policymakers, and researchers aiming to leverage digital tools for maternal and neonatal health equity.

## Supplementary material

10.2196/75738 Multimedia Appendix 1 Search strategies.

10.2196/75738 Multimedia Appendix 2 Critical appraisal of the selected studies using the Mixed Methods Appraisal Tool (MMAT).

10.2196/75738 Multimedia Appendix 3 Digital health interventions used in emergency obstetric and newborn care (EmONC; n=21).

10.2196/75738 Checklist 1 PRISMA-ScR checklist.

## Abbreviations

AI: artificial intelligence
ASDIT: Accessing Safe Deliveries in Tanzania
BIMA: Blood Information Management Application
CDISAH: Classification of Digital Interventions, Services, and Applications in Health
CHW: community health worker
CUG: Closed User Group
DELTA: Digital EmONC Learning and Training Assistant
DHI: digital health intervention
eHBB: electronic Helping Babies Breathe
EmONC: emergency obstetric and newborn care
EOC: essential obstetric care
LMIC: low- and middle-income country
mHealth: mobile health
PIERS-POM: Pre-eclampsia Integrated Estimate of Risk on the Move
PRISMA-ScR: Preferred Reporting Items for Systematic Reviews and Meta-Analyses extension for Scoping Reviews
RCT: randomized controlled trial
SDA: Safe Delivery App
UN IGME: United Nations Inter-Agency Group for Child Mortality Estimation
USSD: Unstructured Supplementary Service Data
VR: virtual reality
WHO: World Health Organization
