# Supplementary material for: Digital Health Interventions in Emergency Obstetric and Newborn Care Services in Low- and Middle-Income Countries: Scoping Review
Source: J Med Internet Res. 2025 Oct 28;27:e75738. doi: 10.2196/75738 (PMC12560964; doi:10.2196/75738)
Supplement: Multimedia Appendix 1 [file jmir-v27-e75738-s001.docx]

# Search strategy.

**Medline PubMed**

| **No** | **Search concept** | **Search terms** |
| --- | --- | --- |
| #1 | Digital health intervention (DHI) | “digital health intervention*” OR ehealth OR e-health OR "electronic health" OR "digital health" OR "digital technolog*" OR "digital intervention*" OR "electronic care" OR telemedicine OR "tele-medicine" OR telehealth OR “tele-health” OR telecare OR “tele-care” OR telemonitoring OR “tele-monitoring” OR teleconsultation OR “tele‐consultation” OR videoconsult* OR "video consult" OR "text messag*" OR texting OR "mobile-health" OR "mobile-care" OR mhealth OR "m-health" OR android OR app OR apps OR audio* OR “cell-phone” OR cellphone OR computer* OR mobile OR multi‐media OR multimedia OR “personal digital assistant” OR PDA OR SMS OR "social medi*" OR software OR application* OR telecomm* OR “e‐Portal” OR ePortal OR eTherap* OR e‐therap* OR forum* OR “information technolog*” OR "instant messag*" OR internet* OR ipad OR i‐pad or iphone OR i‐phone OR ipod OR i‐pod OR android OR web* OR "smart phone" OR smartphone OR “mobile phone” OR e‐mail* OR email* |
| #2 | DHI MeSH | "Medical Informatics"[Mesh] OR "Telemedicine"[Mesh] OR "Digital Health"[Mesh] |
| #3 | DHI Combined | #1 OR #2 |
| #4 | Emergency Obstetric and Neonatal Care (EmONC) | “Emergency Obstetric and Neonatal Care” OR EmONC OR “Emergency Obstetric Care” OR EmOC OR “Emergency Neonatal Care” OR EmNC OR “Emergency obstetric*” OR “Emergency Neonat*” |
| #5 | EmONC MeSH | ("Emergency Medical Services"[Mesh] OR "Emergencies"[Mesh]) AND ("Infant, Newborn"[Mesh] OR "Obstetric Labor Complications"[Mesh]) |
| #6 | EmONC combined | #4 OR #5 |
| #7 | Low- and middle-income countries | “Low and middle income countr*” OR LMIC* OR “low income” OR “lower income” OR “Low income countr*” OR “Lower income countr*” OR “middle income” OR “middle income countr*” OR “lower middle income” OR “lower middle income countr*” OR “upper middle income countr*” OR “poor countr*” OR “developing countr*” OR “developing nation*” OR “developing population*” OR “developing world” OR “less developed countr*” OR “less developed nation*” OR “less developed world” OR “lesser developed countr*” OR “lesser developed nation*” OR “under developed countr*” OR “under developed nation*” OR “under developed world” OR “underdeveloped countr*” OR “underdeveloped nation*” OR “underdeveloped world” OR “middle income nation*” OR “middle income population*” OR “low income nation*” OR “low income population*” OR “lower income nation*” OR “lower income population*” OR “underserved countr*” OR “underserved nation*” OR “underserved population*” OR “under served population*” OR “deprived countr*” OR “deprived population*” OR “poor countr*” OR “poor nation*” OR “poor population*” OR “poor world” OR “poorer countr*” OR “poorer nation*” OR “poorer population*” OR “third world” |
| #8 | L-MIC Regions | "Africa South of the Sahara"[MeSH Terms] OR "asia, central"[MeSH Terms] OR "asia, northern"[MeSH Terms] OR "asia, southern"[MeSH Terms] OR "africa, northern"[MeSH Terms] |
| #9 | Countries | afghan* OR africa OR albania OR algeria OR angola OR argentina OR armenia OR azerbaijan OR bangladesh OR bengal* OR bangal* OR belarus* OR belorus* OR byelarus* OR byelorus* OR belize OR benin OR dahomey OR bhutan OR bolivia OR bosnia OR herzegovin OR botswan* OR batswan* OR bechuanaland* OR brazil* OR brasil* OR bulgaria OR burkina* OR upper volta OR burundi OR urundi OR cabo verde* OR cape verde* OR cambodia OR kampuchea OR khmer OR cameroon OR cameroun OR ubangi shari OR chad OR china OR chinese OR colombia OR comoro OR comore OR comorian OR mayotte OR congo OR zaire OR costa rica OR "cote d'ivoir*" OR "cote d' ivoir*" OR cote divoir* OR cote d ivoir* OR ivory coast* OR ivorian* OR cuba OR cuban OR cubans OR "cuba's" OR djibouti OR french somaliland* OR dominica* OR ecuador OR egypt OR united arab republic* OR el salvador* OR salvadoran* OR guinea OR equatoguinea* OR eritrea OR eswatini OR swaziland OR swazi* OR swati* OR ethiopia OR fiji OR gabon* OR gabonese* OR gabonaise* OR gambia OR ghana OR grenada OR grenadian OR guatemala OR haiti OR hispaniola OR hondura* OR india OR indonesia OR iran* OR iraq OR jamaica OR jordan* OR kazakh* OR kenya OR karabati OR korea OR Kosovo OR kosova OR kyrgyz* OR kirgiz* OR kirghiz* OR laos OR lao OR laotian OR lebanon OR lebanese OR lesotho OR basutoland OR mosotho OR basotho OR liberia OR libya OR jamahiriya OR macedonia OR madagasca* OR malagasy* OR malawi OR nyasaland* OR malaysia OR maldives OR maldivian OR indian ocean OR mali OR malian OR "mali's" OR micronesia OR marshallese OR kiribati OR marshall island OR palau OR paluan OR tuvalu OR mauritan* OR mauritius OR mexico OR mexican* OR moldova OR moldovia OR mongol* OR montenegr* OR morocco OR moroccan* OR ifni OR mozambique OR mozambican OR myanmar OR burma OR burmese OR namibia OR nepal OR antill* OR nicaragua OR niger* OR pakistan OR palestin* OR gaza OR west bank OR paraguay OR peru OR peruvian OR "peru's" OR philippine OR philipine OR phillipine OR phillippine OR filipino OR filipina OR russia OR ussr* OR soviet OR rwanda OR rwandese OR ruanda OR ruandese OR samoa OR navigator island* OR pacific island* OR "sao tome and principe*" OR sao tomean OR santomean OR senegal OR serbia OR sierra leone OR south africa OR melanesia OR solomon island* OR norfolk island* OR somali* OR sri lanka OR ceylon OR saint lucia OR st lucia OR saint vincent OR st vincent OR vincentian OR grenadine OR sudan OR surinam* OR syria OR tajik OR tadjik OR tadzhik OR tanzania OR tanganyika OR thai* OR timor leste OR east timor OR timorese OR togo OR "togo's" OR tonga OR tunisia OR turkiy* OR turkey OR turk OR turks OR turkish OR turkmen* OR uganda OR ukrain* OR uzbek* OR vanuatu OR new hebrides* OR venezuela OR vietnam* OR viet nam OR yemen OR yugoslav* OR zambia OR Zimbabwe |
| #10 | L-MIC Combined | #7 OR #8 OR #9 |
| #11 | Combined searches | #3 AND #6 AND #10 |

## **Cochrane library**

| **No** | **Search concept** | **Search terms** |
| --- | --- | --- |
| #1 | **Digital health intervention (DHI)** | (digital NEXT health NEXT intervention*) OR ehealth OR e-health OR "electronic health" OR "digital health" OR (digital NEXT technolog*) OR (digital NEXT intervention*) OR "electronic care" OR telemedicine OR "tele-medicine" OR telehealth OR “tele-health” OR telecare OR “tele-care” OR telemonitoring OR “tele-monitoring” OR teleconsultation OR tele‐consultation OR videoconsult* OR "video consult" OR (text NEXT messag*) OR texting OR "mobile-health" OR "mobile-care" OR mhealth OR "m-health" OR android OR app OR apps OR audio* OR “cell-phone” OR cellphone OR computer* OR mobile OR multi‐media OR multimedia OR “personal digital assistant” OR PDA OR SMS OR (social NEXT medi*) OR software OR application* OR telecomm* OR “e‐Portal” OR ePortal OR eTherap* OR (e NEXT therap*) OR forum* OR (information NEXT technolog*) OR (instant NEXT messag*) OR internet* OR ipad OR i‐pad or iphone OR i‐phone OR ipod OR i‐pod OR android OR web* OR "smart phone" OR smartphone OR “mobile phone” OR e‐mail* OR email* |
| #2 | **Digital health**  [MeSH term search] | Digital health |
| #3 | **Telemedicine**  [MeSH term search] | Telemedicine |
| #4 | **Health Informatics**  [MeSH term search] | Health Informatics |
| #5 | **DHI combined** | #1 OR #2 OR #3 OR #4 |
| #6 | **Emergency Obstetric and Neonatal Care (EmONC)** | “Emergency Obstetric and Neonatal Care” OR EmONC OR “Emergency Obstetric Care” OR EmOC OR “Emergency Neonatal Care” OR EmNC OR (Emergency NEXT obstetric*) OR (Emergency NEXT Neonat*) |
| #7 | **EmONC**  [MeSH term search] | Infant, Newborn |
| #8 | **EmONC**  [MeSH term search] | Obstetric Labor Complications |
| #9 | **EmONC**  [MeSH term search] | Emergency Medical Services |
| #10 | **EmONC MeSH combined** | (#7 OR #8) AND #9 |
| #11 | **EmONC combined** | #6 OR #10 |
| #12 | **Sub-Saharan Africa**  [MeSH term search] | Africa South of the Sahara |
| #13 | **Central Asia**  [MeSH term search] | Asia, Central |
| #14 | **Northern Asia**  [MeSH term search] | Asia, Northern |
| #15 | **Southern Asia**  [MeSH term search] | Asia, Southern |
| #16 | **Northern Africa**  [MeSH term search] | Africa, Northern |
| #17 | **L-MIC Region MeSH combined** | #12 OR #13 OR #14 OR #15 OR #16 |
| #18 | **L-MIC** | developing countr* OR developing nation* OR developing population* OR developing world OR less developed countr* OR less developed nation* OR less developed world OR lesser developed countr* OR lesser developed nation* OR lesser developed world OR under developed countr* OR under developed nation* OR under developed world OR underdeveloped countr* OR underdeveloped nation* OR underdeveloped world OR middle income countr* OR middle income nation* OR middle income population* OR low income countr* OR low income nation* OR low income population* OR lower income countr* OR lower income nation* OR lower income population* OR underserved countr* OR underserved nation* OR underserved population* OR under served population* OR under served nation* OR under served population* OR deprived countr* OR deprived population* OR high burden countr* OR high burden nation* OR countdown countr* OR countdown nation* OR poor countr* OR poor nation* OR poor population* OR poor world OR poorer countr* OR poorer nation* OR poorer population* OR poorer world OR lmic OR lmics OR third world |
| #19 | **Countries** | afghan* OR africa* OR albania* OR algeria* OR angola* OR argentin* OR armenia* OR azerbaijan* OR bangladesh* OR bengal* OR bangal* OR belarus* OR belorus* OR byelarus* OR byelorus* OR belize* OR benin* OR dahomey OR bhutan* OR bolivia* OR bosnia* OR herzegovin* OR botswan* OR batswan* OR bechuanaland* OR brazil* OR brasil* OR bulgaria* OR burkina* OR burkinese* OR upper volta* OR burundi* OR urundi* OR cabo verde* OR cape verde* OR cambodia* OR kampuchea* OR khmer* OR cameroon* OR cameroun* OR (ubangi NEXT shari*) OR chad* OR china* OR chinese OR colombia* OR comoro* OR comore* OR comorian* OR mayotte* OR congo* OR zaire* OR (costa NEXT rica*) OR (cote NEXT d'ivoir*) OR (cote NEXT divoir*) OR (ivory NEXT coast*) OR ivorian* OR cuba OR cuban OR cubans OR "cuba's" OR djibouti* OR (french NEXT somaliland*) OR dominica* OR ecuador* OR egypt* OR united arab republic OR (el NEXT salvador*) OR salvadoran* OR guinea* OR equatoguinea* OR eritrea* OR eswatini* OR swaziland* OR swazi* OR swati* OR ethiopia* OR fiji* OR gabon* OR gabonese* OR gabonaise* OR gambia* OR ghana* OR grenada* OR grenadian* OR guatemala* OR haiti* OR hispaniola* OR hondura* OR india* OR indonesia* OR iran* OR iraq* OR jamaica* OR jordan* OR kazakh* OR kenya* OR karabati* OR korea* OR kosovo* OR kosova* OR kyrgyz* OR kirgiz* OR kirghiz* OR laos OR lao OR laotian* OR lebanon* OR lebanese* OR lesotho* OR lesothan* OR lesothonian* OR basutoland* OR mosotho* OR basotho* OR liberia* OR libya* OR jamahiriya* OR macedonia* OR madagasca* OR malagasy* OR malawi* OR nyasaland* OR malaysia* OR maldives* OR maldivian* OR indian ocean OR mali OR malian* OR "mali's" OR micronesia* OR marshallese* OR kiribati* OR (marshall NEXT island*) OR palau OR paluan* OR tuvalu* OR mauritania* OR mauritan* OR mauritius* OR mexico* OR mexican* OR moldova* OR moldovia* OR mongol* OR montenegr* OR morocco* OR moroccan* OR ifni OR mozambique* OR mozambican* OR myanmar* OR burma* OR burmese OR namibia* OR nepal* OR antill* OR nicaragua* OR niger* OR pakistan* OR palestin* OR gaza* OR west bank* OR paraguay* OR peru OR peruvian* OR "peru's" OR philippine* OR philipine* OR phillipine* OR phillippine* OR filipino* OR filipina* OR russia* OR ussr* OR soviet* OR rwanda* OR rwandese OR ruanda* OR ruandese OR samoa* OR (navigator NEXT island*) OR (pacific NEXT island*) OR "sao tome and principe" OR (sao NEXT tomean*) OR santomean* OR senegal* OR serbia* OR sierra leone OR south africa OR melanesia* OR (solomon NEXT island*) OR (norfolk NEXT island*) OR somali* OR sri lanka OR ceylon* OR saint lucia OR st lucia OR saint vincent OR st vincent OR vincentian* OR grenadine* OR sudan* OR surinam* OR syria* OR tajik* OR tadjik* OR tadzhik* OR tanzania* OR tanganyika* OR thai* OR timor leste OR east timor OR timorese* OR togo OR togoles* OR "togo's" OR tonga* OR tunisia* OR turkiy* OR turkey* OR turk OR turks OR turkish OR turkmen* OR uganda* OR ukrain* OR uzbek* OR vanuatu* OR (new NEXT hebrides*) OR venezuela* OR vietnam* OR viet nam* OR yemen* OR yugoslav* OR zambia* OR Zimbabwe* |
| #20 | **LMIC Combined** | #17 OR #18 OR #19 |
| #21 | **All combined** | #5 AND #11 AND #20 |

**Web of Science**

| **No** | **Search concept** | **Search terms** |
| --- | --- | --- |
| #1 | **Digital health intervention (DHI)** | “digital health intervention*” OR ehealth OR e-health OR "electronic health" OR "digital health" OR "digital technolog*" OR "digital intervention*" OR "electronic care" OR telemedicine OR "tele-medicine" OR telehealth OR “tele-health” OR telecare OR “tele-care” OR telemonitoring OR “tele-monitoring” OR teleconsultation OR tele‐consultation OR videoconsult* OR "video consult" OR "text messag*" OR texting OR "mobile-health" OR "mobile-care" OR mhealth OR "m-health" OR android OR app OR apps OR audio* OR “cell-phone” OR cellphone OR computer* OR mobile OR multi‐media OR multimedia OR “personal digital assistant” OR PDA OR SMS OR "social medi*" OR software OR application* OR telecomm* OR “e‐Portal” OR exportal OR eTherap* OR e‐therap* OR forum* OR “information technolog*” OR "instant messag*" OR internet* OR ipad OR i‐pad or iphone OR i‐phone OR ipod OR i‐pod OR android OR web* OR "smart phone" OR smartphone OR “mobile phone” OR e‐mail* OR email* (Topic) |
| #2 | **EmONC** | “Emergency Obstetric and Neonatal Care” OR EmONC OR “Emergency Obstetric Care” OR EmOC OR “Emergency Neonatal Care” OR emic OR “Emergency obstetric*” OR “Emergency Neonat*” (Topic) |
| #3 | **LMIC Countries** | afghan* OR africa OR albania OR algeria OR angola OR argentina OR armenia OR azerbaijan OR bangladesh OR bengal* OR bangal* OR belarus* OR belorus* OR byelarus* OR byelorus* OR belize OR benin OR dahomey OR bhutan OR bolivia OR bosnia OR herzegovina OR botswan* OR batswan* OR bechuanaland* OR brazil* OR brasil* OR bulgaria OR burkina* OR burkinese* OR upper volta OR burundi OR ulundi OR cabo verde* OR cape verde* OR cambodia OR kampuchea OR khmer OR cameroon OR cameroun OR usangi shari OR chad OR china OR chinese OR colombia OR comoro OR comore OR comorians OR mayotte OR congo OR zaire OR costa rica OR "cote d'ivoir*" OR "cote d' ivoir*" OR cote divoir* OR cote d ivoir* OR ivory coast* OR ivorian* OR cuba OR cuban OR cubans OR "cuba's" OR djibouti OR french somaliland* OR dominica* OR ecuador OR egypt OR united arab republic* OR el salvador* OR salvadoran* OR guinea OR equatoguinea* OR eritrea OR eswatini OR swaziland OR swazi* OR swati* OR ethiopia OR fiji OR gabon* OR gabonese* OR gabonaise* OR gambia OR ghana OR grenada OR grenadian OR guatemala OR haiti OR hispaniola OR hondura* OR india OR indonesia OR iran* OR iraq OR jamaica OR jordan* OR kazakh* OR kenya OR karabali OR korea OR Kosovo OR kosova OR kyrgyz* OR kirgiz* OR kirghiz* OR laos OR lao OR laotian OR lebanon OR lebanese OR lesotho OR lessthan OR lesothoan OR basutoland OR mosocho OR basotho OR liberia OR libya OR jamahiriyah OR macedonia OR madagasca* OR malagasy* OR malawi OR nyasaland* OR malaysia OR maldives OR maldivian OR indian ocean OR mali OR malian OR "mali's" OR micronesia OR marshallese OR kiribati OR marshall island OR palau OR papuan OR tuvalu OR mauritan* OR mauritius OR mexico OR mexican* OR moldova OR moldavia OR mongol* OR montenegr* OR morocco OR moroccan* OR ifni OR mozambique OR mozambican OR myanmar OR burma OR burmese OR namibia OR nepal OR antill* OR nicaragua OR niger* OR pakistan OR palestin* OR gaza OR west bank OR paraguay OR peru OR peruvian OR "peru's" OR philippine OR philippine OR phillipines OR phillippines OR filipino OR filipina OR russia OR ussr* OR soviet OR rwanda OR randes OR ruanda OR rwandese OR samoa OR navigator island* OR pacific island* OR "sao tome and principe*" OR sao tnmean OR santomea OR senegal OR serbia OR sierra leone OR south africa OR melanesia OR solomon island* OR norfolk island* OR somali* OR sri lanka OR ceylon OR saint lucia OR st lucia OR saint vincent OR st vincent OR vincentians OR grenadines OR sudan OR surinam* OR syria OR tajik OR tajik OR tadzhika OR tanzania OR tanganyika OR thai* OR timor leste OR east timor OR timorese OR togo OR toggles OR "togo's" OR tonga OR tunisia OR turkiy* OR turkey OR turk OR turks OR turkish OR turkmen* OR uganda OR ukrain* OR uzbek* OR vanuatu OR new hebrides* OR venezuela OR vietnam* OR viet nam OR yemen OR yugoslav* OR zambia OR Zimbabwe (Topic) |
| #4 | **Combined** | #1 AND #2 AND #3 |
