# Supplementary material for: Digital Health Interventions in Emergency Obstetric and Newborn Care Services in Low- and Middle-Income Countries: Scoping Review
Source: J Med Internet Res. 2025 Oct 28;27:e75738. doi: 10.2196/75738 (PMC12560964; doi:10.2196/75738)
Supplement: Multimedia Appendix 3 [file jmir-v27-e75738-s003.docx]

Digital health interventions used in emergency obstetric and newborn care (EmONC; n=21).

| **WHO Class** | **DHI name** | **Purpose** | **Delivery Mode** | **Features and Functionalities of DHIs** |
| --- | --- | --- | --- | --- |
| **DHIs targeting pregnant women** | | | | |
| 1.5 Person-based reporting | Text4Life app [35] | To facilitate real-time, two-way communication between a woman facing pregnancy complications and healthcare providers coordinating emergency transportation. | Phone calls/text messages,  a mobile application | 1. A pregnant woman activated an alert system by sending an SMS to a phone number linked to a central server, where patient information is stored using a web user interface called “Textit” 2. The server automatically responded, instructing the woman to wait. 3. At the same time, a dual SMS containing the woman's name and address was sent to a community committee and a primary healthcare centre, notifying them of the emergency. |
| **DHIs targeting healthcare providers** | | | | |
| 2.2 Person-centred health records  2.3 Healthcare provider decision support* | Digital EmONC register [36] | To digitalise data registry, enhance the quality of data, and provide real-time access to EmONC-related information in healthcare facilities. | E-register application | 1. The e-register application captured all 50 variables in a sequence mirroring the paper-based register. It also included decision prompts, error-checking features, and guidance on patient care for healthcare providers. 2. The integrated web-based dashboard provides graphical presentations of real-time facility data. 3. Tablet computers containing the application were placed in labour rooms and operating theatres at the implementation facilities, replacing paper-based registers. |
| 2.2 Person-centred health records 2.4 Tele-medicine* | Call centre [37] | To capture patient-reported 7-days post-delivery outcomes (mothers and newborns). | A call centre | 1. The call centre utilised a questionnaire to monitor mothers' and newborns' post-delivery conditions and stored the data securely in a cloud-based server. 2. If a health issue was identified during the call, the call centre staff referred the patients to a nearby health facility. |
| 2.2 Person-centred health records  2.6 Referral coordination* | RapidSMS – Maternal and Child Health (MCH) [38] | To facilitate two-way communication between community health workers (CHWs) and the rest of the health system, which also kept a database of clinical records of maternal care delivery. | Phone calls/text messages | 1. CHWs logged new pregnancies into the RapidSMS-MCH system, which then sent reminders about antenatal care appointments and delivery dates to the CHWs. 2. Danger signs of mothers/infants which were reported would activate an emergency alert system and gave immediate feedback to the CHWs. Ambulance drivers and facility managers would get information about the situation and the CHW’s contact details.  3. A password-protected web interface provided an overview of the system's outputs and reports. |
| 2.3 Healthcare provider decision support | mHealth Clinical Decision-Making Support Intervention (mCDMSI) [39] | To provide clinical decision-making support for frontline healthcare providers that aimed to improve neonatal mortality. | Phone calls/text messages | 1. Emergency protocols were accessible for healthcare providers through Unstructured Supplementary Service Data (USSD), and additional support was available via voice calls, SMS, and internet access. 2. Each mobile phone assigned to the project was equipped with a unique Subscriber Identification Module (SIM) card. All SIM cards were part of a Closed User Group (CUG), enabling free and unlimited USSD access. |
|  | Pre-eclampsia Integrated Estimate of Risk (PIERS)- On-the-Move (POM) app combined with Community-Level Interventions for Pre-eclampsia (CLIP) [40] | To provide community engagement and CHWs-led app-guided triage, treatment, and transport to facilities for pregnant women with hypertension. | A mobile application | 1. CHWs received training to provide hypertension-in-pregnancy care during home visits, utilising the PIERS-POM app for risk assessment. 2. The app provided guidance on administering oral methyldopa or intramuscular magnesium sulphate or referring women to comprehensive emergency obstetric care facilities. |
|  | Artificial Intelligence (AI) augmented Continuous Electronic Fetal Monitoring (CEFM) [41] | To provide an automated early warning system and clinical decision support tool during labour and delivery. | A laptop,  PeriWatch Vigilance® software | 1. Fetal monitors were connected to patients’ bedsides, and PeriWatch Vigilance® continuously tracked maternal vital signs, fetal heart rate, contractions, and labour progression. 2. Data collected at the bedside was sent to a server in Houston, where PeriGen’s AI analytics applied a risk stratification algorithm to analyse it.  3. Obstetrician services, either on-site or remote, were available 24/7. 4. The assessments were transmitted back to the bedside within seconds, allowing clinicians to access the maternal-fetal early warning system directly from various devices, including desktops, tablets, and mobile phones. |
| 2.4 Tele-medicine | Telehealth combined with an educational model [42] | To provide communication and educational model between two hospitals for collaboratively addressing obstetric emergencies and enhancing maternal and perinatal outcomes. | Video conferencing tools,  Liliconnect (a platform to exchange patients' records) | 1. Healthcare providers from the second-level hospital received educational modules and workshops on obstetric emergencies, along with continuous support via WhatsApp chat with staff from the tertiary hospital. 2. Pregnant women with obstetric emergencies were evaluated via telemedicine services. 3. Teleconferences between two hospitals were conducted at least once every three months to discuss telehealth implementation, compliance, and any difficulties. |
| 2.6 Referral coordination | Call centre [43] | To improve the referral processes for maternal and newborn emergencies and provide reliable expert advice to frontline healthcare providers. | A call centre | 1. The service was promoted exclusively to frontline healthcare providers making referrals and the hospitals receiving those referrals. 2. Frontline service providers could contact experts (obstetricians, paediatricians, the regional pharmacist, and an anaesthetist) via a phone call routed through the centre. 3. If a referral was needed, based on advice from the expert panel or feedback from the health facility, the follow-up officers arranged the referral. |
|  | Kampala Digital Emergency Transport System (KDETS) mobile app combined with Emergency Call and Dispatch Centre (ECDC) [44] | To initiate the referral process, deploy and track ambulances, and provide feedback for the referral systems. | A mobile application,  a call centre | 1. Health facilities used the KDETS app to request referral transport. 2. Ambulance requests were communicated via the ECDC. 3. ECDC agents dispatched the pickup request to available ambulance drivers and tracked their location using ambulance tracking screens. 4. ECDC agents notified the receiving facility's providers to ensure they were prepared. 5. Ambulance drivers could input additional information on the app to help monitor response time data. |
|  | Mobile Obstetric Emergency System (MORES) [45] | To improve the referral processes for maternal and newborn emergencies. | Text messages | 1. Interactive obstetric triage and MORES referral training sessions were held, which included an overview of MORES, assignment of unique IDs, and guidance on messaging templates for both referring and receiving facilities. 2. Completed templates were shared exclusively within the specific WhatsApp group connecting each RHF with its corresponding district hospital. |
| 2.8 Healthcare provider training | Life-Saving Instruction for Emergencies (LIFE) app [46] | To provide training in caring for critically ill newborns and children. | A mobile application | 1. The LIFE app enhances the scenario-based Emergency Triage, Assessment, and Treatment Plus admission care (ETAT+) training model. 2. Adaptive or standardized immediate feedback was given after each incorrect attempt at a learning task.  3. Three progressively detailed levels of feedback were provided, determined by the predicted likelihood of the learner's next attempt being correct. 4. Upon successfully completing a learning session, the platform generated a performance score reflecting whether the learner's responses to the tasks were correct on the first attempt. |
|  | Safe Delivery App (SDA) [47–54] | To provide EmONC training for healthcare providers in rural areas. | A mobile application | 1. The SDA is available for free download in various language versions. The app included videos demonstrating basic EmONC procedures, each detailing specific steps. The app was designed with features suitable for low-literacy and low-income settings and could function entirely offline once downloaded. 2. The app also had a catalogue of essential drugs and equipment for basic EmONC. 3. Users received daily notifications to promote app usage, along with weekly notifications containing quiz questions and direct links to videos that provide information necessary to answer the questions. 4. The app included a tracking feature that recorded the usage of various content along with GPS coordinates. |
|  | Shorter, blended-learning approach (face-to-face classroom combined with online instruction) [55] | To enhance traditional EmONC training while significantly reducing costs. | Phone calls/text messages | 1. The blended learning approach included a shorter offsite training phase followed by onsite learning through SMS messages and phone calls. 2. A server was programmed to send daily SMS messages containing EmONC training materials to providers' mobile phones. Some of these messages included multiple-choice questions requiring a response. 3. Upon receiving the provider's response, an automated feedback system sent another message confirming the correct answer and providing further explanations. 4. Providers who did not respond to SMS messages were monitored using an SMS tracking sheet and encouraged to participate. 5. In addition to SMS messages, healthcare providers received weekly phone calls discussing challenging cases encountered in real clinic settings. |
|  | PRONTO simulation and team training combined with Emergency Maternal and Neonatal Care Preparedness (AMANAT) [56–60] | To enhance healthcare providers’ competency in delivering essential intrapartum and newborn care services. | Videos, PartoPants™, NeoNatalie® simulators | 1. AMANAT is a mobile nurse mentoring program. Each month, a pair of nurse-mentors visited a facility for one week before moving on to the next, covering all four assigned facilities in rotation. They returned to each facility for week-long visits every month for eight months. 2. PRONTO simulation and team training were integrated into the AMANAT program. The simulations featured a maternal actor wearing PartoPants™ for vaginal delivery, and a NeoNatalie® infant mannequin. 3. During each site visit, mentors were required to conduct simulations of three clinical scenarios, which included vaginal deliveries and neonatal resuscitation. 4. All mentors were trained to conduct and video record simulations, facilitate video-guided debriefings after simulations, and lead debriefings following live births. |
|  | PRONTO simulation and team training combined with The East Africa Preterm Birth Initiative (PTBi-EA) [61] | To enhance healthcare providers’ competency in delivering essential intrapartum and newborn care services. | Videos, PartoPants™, NeoNatalie® simulators,  StudioCode™ software | 1. The PTBi-EA package comprised enhanced data collection, an adapted WHO Safe Childbirth Checklist, a quality improvement collaborative, and a modified version of PRONTO training. 2. PRONTO incorporates video recording, enabling training examiners to review the footage later to identify practice issues and give feedback to participants meticulously. 3. Throughout each training session, simulations were video recorded and then sent to a trained video analyst (VA) in each country, who was a clinician but not a PRONTO mentor. 4. The VAs uploaded the recordings into the StudioCode™ software to identify the practices appropriate for each simulation. They reviewed the videos using the software and coded instances when an Evidence-Based Practice (EBP) was observed. |
|  | electronic Helping Babies Breathe (eHBB) app and Virtual Reality (VR) simulations [62] | To maintain neonatal resuscitation skills in healthcare workers. | A mobile application,  a VR headset (Google Cardboard) | 1. The eHBB VR app includes simulation scenarios aligned with the HBB curriculum to enhance in-person training sessions. 2. HCWs interacted with the three-dimensional objects in the simulation by either pressing a button-lever on the outside of the headset or by tapping the screen. 3. Trainers used the mobile Helping Babies Survive app powered by the District Health Information System (mHBS/DHIS2) tracker app, which included digital versions of the HBB knowledge check, BMV skill check, and OSCE A and B checklists. 4. Participants used the mHBS/DHIS2 tracker app to document their HBB corner practice.  5. These apps can be used without an internet connection. |
|  | Digital EmONC Learning and Training Assistant (DELTA) combined with mentoring programme [63] | To provide a free self-guided digital learning platform for healthcare providers working in maternity or related service areas. | A mobile application | 1. The facility-based mentors received EmONC training based on Kenya's curriculum to demonstrate skills, facilitate skills drills training, and provide feedback to mentees. 2. More EmONC training materials were transferred to mentee via WhatsApp. 3. The mentors logged weekly activities with mentees on a ‘Mentor Tracker Platform’ using an open-source mobile application. 4. Both mentors and mentees had access to 12 EmONC modules on the platform at any time. |
| 2.8 Healthcare provider training  2.4 Tele-medicine* | Accessing Safe Deliveries in Tanzania (ASDIT) [64] | To expand access to comprehensive EmONC services in underserved rural areas by leveraging existing human resources at healthcare facilities. | Phone calls/text messages | 1. Face-to-face training sessions covered Comprehensive EmONC and anaesthesia. 2. Following the training, capacity building included teleconsultations, quarterly onsite supportive supervision, and ongoing mentorship via telephone and social media to reinforce skills and knowledge. Health centre providers were connected with obstetricians for virtual consultations during maternal complications.  3. Clinical audits were conducted for all mothers who died or had significant morbidities. |
|  | M-communication combined with Non-pneumatic Anti-Shock Garment (NASG) [65] | To provide a communication network and training resources for healthcare providers for using the NASG during obstetric haemorrhage. | Phone calls/text messages | 1. The NASG was introduced alongside a CUG mobile phone network. 2. Phones on the CUG network could call other phones within the network without airtime charges, enabling healthcare providers to communicate and seek patient support. 3. The CUG phones were also equipped with a data entry system created on OpenXdata, accessible remotely via a secure web-based platform. 4. Training sessions specific to the NASG were conducted, supported by training videos uploaded to the CUG phones during supervision, to refresh trained staff or train new staff. |
| **DHIs targeting health systems** | | | | |
| 3.2 Supply Chain Management  B.1 Blood bank information management systems | Blood Information Management Application (BIMA) [66,67] | To reduce the wait time for emergent obstetric patients needing blood transfusions. | An online blood information application system | 1. The BIMA system recorded details about the names and locations of blood centres, current blood unit stock, donor lists (including blood group and Rh typing), availability of specific blood groups, and booking information for needed blood. 2. The system linked the obstetric wards with nearby blood banks located within the hospital premises. This digital system updated the blood availability records each morning and worked closely with both the ward and the blood bank. |

*Additional class function
